# Quantifying resilience of multiple ecosystem services and biodiversity in a temperate forest landscape

**DOI:** 10.1002/ece3.3491

**Published:** 2017-10-16

**Authors:** Elena Cantarello, Adrian C. Newton, Philip A. Martin, Paul M. Evans, Arjan Gosal, Melissa S. Lucash

**Affiliations:** ^1^ Department of Life and Environmental Sciences Bournemouth University Poole UK; ^2^ Department of Environmental Science and Management, SRTC B1‐04D Portland State University Portland OR USA

**Keywords:** biodiversity, climate change impacts, dieback, disturbance, ecosystem services, forest collapse, forest management, grazing, LANDIS‐II, multiple stressors, socio‐ecological resilience

## Abstract

Resilience is increasingly being considered as a new paradigm of forest management among scientists, practitioners, and policymakers. However, metrics of resilience to environmental change are lacking. Faced with novel disturbances, forests may be able to sustain existing ecosystem services and biodiversity by exhibiting resilience, or alternatively these attributes may undergo either a linear or nonlinear decline. Here we provide a novel quantitative approach for assessing forest resilience that focuses on three components of resilience, namely resistance, recovery, and net change, using a spatially explicit model of forest dynamics. Under the pulse set scenarios, we explored the resilience of nine ecosystem services and four biodiversity measures following a one‐off disturbance applied to an increasing percentage of forest area. Under the pulse + press set scenarios, the six disturbance intensities explored during the pulse set were followed by a continuous disturbance. We detected thresholds in net change under pulse + press scenarios for the majority of the ecosystem services and biodiversity measures, which started to decline sharply when disturbance affected >40% of the landscape. Thresholds in net change were not observed under the pulse scenarios, with the exception of timber volume and ground flora species richness. Thresholds were most pronounced for aboveground biomass, timber volume with respect to the ecosystem services, and ectomycorrhizal fungi and ground flora species richness with respect to the biodiversity measures. *Synthesis and applications*. The approach presented here illustrates how the multidimensionality of stability research in ecology can be addressed and how forest resilience can be estimated in practice. Managers should adopt specific management actions to support each of the three components of resilience separately, as these may respond differently to disturbance. In addition, management interventions aiming to deliver resilience should incorporate an assessment of both pulse and press disturbances to ensure detection of threshold responses to disturbance, so that appropriate management interventions can be identified.

## 1 INTRODUCTION

Forests have evolved in the presence of natural disturbances, such as drought, windstorms, wildfire, insect, and disease outbreaks (Greenberg & Collins, 2015; Walker, 1999). However, the increasing frequency, extent, and severity of disturbances are altering forest communities outside the ranges within which they have evolved and adapted (Usbeck et al., 2010; Weed, Ayres, & Hicke, 2013). As a result of the current high rate of global environmental change, the intensification of forest disturbances is likely to continue, which may inhibit the ability of species to keep pace through their evolutionary adaptation processes (Trumbore, Brando, & Hartmann, 2015). As a result, the future of global forests, their associated biodiversity, and the provision of ecosystem services to human society are uncertain (Trumbore et al., 2015). These services include provisioning (e.g., timber), regulating (e.g., carbon sequestration), supporting (e.g., nutrient cycling), and cultural (e.g., recreation) benefits (MEA 2005). When expressed in monetary units, these combined services have been estimated to be worth 5,264 and 3,013 international $/ha/year in tropical and temperate forests, respectively (de Groot et al., 2012). Forests also contain more than 80% of terrestrial species, providing an important source of biodiversity worldwide (FAO 2012). If species are not able to adapt to the intensified disturbances that are widely occurring, the maintenance of biodiversity and the sustainable provisioning of ecosystem services to society could be undermined (Lindner et al., 2010).

Recent research has focused on understanding the trajectory of forest system responses to disturbances, including the role of thresholds and changes in ecological state (Allen, Breshears, & McDowell, 2015). Millar and Stephenson (2015) theorized four patterns of forest response to cumulative disturbances: Response (1) corresponds to a resilient forest, able to sustain existing ecosystem services and where no thresholds are reached; response (2) and (3) both represent a forest crossing a threshold, leading to the conversion to a new forest type. Under (2), the forest is still able to sustain primary ecosystem services, whereas under (3) the changes are substantial enough that ecosystem service delivery declines. Response (4) corresponds to a forest that following the crossing of a threshold transforms to a nonforest type, losing forest function and its capacity to deliver most forest ecosystem services. Given that disturbances are spatially explicit processes playing a key role in forest ecosystem dynamics, landscape approaches are required to determine whether there are abrupt thresholds or more subtle changes in these systems and to evaluate the trajectory of forest recovery (Seidl et al., 2011; Trumbore et al., 2015). Following a disturbance, some forest functions such as photosynthesis and transpiration can recover within a decade, whereas it can take >100 years for biomass and biodiversity to recover (Martin, Newton, & Bullock, 2013; Spake, Ezard, Martin, Newton, & Doncaster, 2015; Trumbore et al., 2015). If we can anticipate an approaching forest transition, guidance can be provided on how management can be adapted so that biodiversity and ecosystem service delivery is maintained.

One response strategy to intensified disturbances is to enhance ecosystem resilience, which has been the focus of recent literature (Biggs et al., 2012), and environmental policy (Newton, 2016; Newton & Cantarello, 2015). Resilience is intuitively understood as the ability of an ecosystem to withstand or tolerate a perturbation. However, the precise definition of resilience in an ecological context has been the focus of substantial debate (Newton & Cantarello, 2015). Many different definitions have been proposed, including engineering resilience (the time required for a system to return to an equilibrium point following a disturbance event; Pimm, 1984) and ecological resilience (the amount of disturbance that a system can absorb before transitioning to another stable state; Brand & Jax, 2007). Promoting resilience through forest management is particularly relevant in the context of intensified disturbances, because the stochastic nature of such disturbances makes them difficult to predict (Seidl, 2014). In addition, disturbances do not act in separation, but can interact in ways that increase their impact. For example, warmer temperatures are expected to amplify the occurrence of pest species, and interactions with drought can further accelerate tree mortality in insect‐damaged trees (Dale et al., 2001).

Despite the emerging importance of resilience as a new paradigm of forest ecosystem management among scientists, practitioners, and policymakers (Millar & Stephenson, 2015; Newton & Cantarello, 2015; Seidl, Spies, Peterson, Stephens, & Hicke, 2016), theoretical discussions of resilience concepts still greatly outpace their practical application (Biggs et al., 2012). This can be attributed to knowledge gaps regarding the underlying mechanisms, and the difficulties in measuring resilience in ways that are appropriate for informing management (Biggs et al., 2012; Reyer et al., 2015). Recent research has identified some approaches that can potentially be used to measure resilience. Methods include rapid assessment approaches (Nemec et al., 2013), the quantification of functional diversity and response diversity (Angeler et al., 2014), discontinuity approaches (Nash, Graham, Jennings, Wilson, & Bellwood, 2016), and thresholds analysis (Standish et al., 2014). However, very few studies have proposed quantifiable metrics, and even in these cases (Nash et al., 2016), they are largely limited to freshwater and marine ecosystems. Potential ways forward for terrestrial ecosystems such as forests include assessment of different elements of resilience, such as resistance and recovery (Newton & Cantarello, 2015; Nimmo, Mac Nally, Cunningham, Haslem, & Bennett, 2015), which should be standardized and compared across systems and fields of research (Hodgson, McDonald, & Hosken, 2015). Measures of resilience should also take into account the spatial and temporal components of disturbances, which are rarely considered (Allen et al., 2016).

Here we provide a novel quantitative assessment approach for assessing the resilience of forest ecosystems that accounts for the spatiotemporal patterns of disturbances and focuses on three measurable resilience components: resistance, recovery, and net change (Nimmo et al., 2015). We explore to what extent a forest that is currently undergoing dieback (Martin, Newton, Cantarello, & Evans, 2015) will be resilient to future disturbances using a spatially dynamic model supported by empirical data, simulating both “pulse” (sudden disturbance) and “press” (sustained disturbance) dynamics, following the conceptual framework presented in Collins et al. (2011). Specifically, we aim to quantify (1) to what extent forest ecosystem services and biodiversity are resistant to pulse and press disturbances; (2) to what extent forest ecosystem services and biodiversity recover from pulse and press disturbances; (3) whether perturbed forest ecosystem services and biodiversity are able to persist over time; and (4) whether there are any thresholds observed in loss of ecosystem service provision and biodiversity when disturbance intensifies over time. Specifically, we tested the hypothesis that all three components of resilience were correlated with each other.

## 2 MATERIALS AND METHODS

### 2.1 Study area

The New Forest National Park is located in southern England (UK; 50°52′00″N 1°34′00″W) and extends over 57,100 ha (Newton, 2010). Its exceptional importance for nature conservation is reflected in its many designations, ranging from national‐scale legislation (e.g., Site of Special Scientific Interest—SSSI), to global‐scale designations (Cantarello, Green, & Westerhoff, 2010). The Park is also one of the most visited in Britain with over 13 million day visits each year (Forestry Commission 2008). The vegetation is composed of ancient pasture‐woodlands, lowland heathland, valley mire communities, acid grassland, the network of rivers and streams, and permanent and temporary ponds. Nowhere else in lowland England do these habitats occur together and at such a large scale (Cantarello et al., 2010). The unique character of the New Forest is strongly dependent on its history as a medieval Royal hunting reserve and the long‐term survival of a traditional commoning system, with large populations of deer and free‐roaming livestock (principally ponies and cattle) interacting with the processes of ecological succession (Newton, Cantarello, Tejedor, & Myers, 2013). The New Forest has been remarkably resilient as a socio‐ecological system having withstood profound political and socioeconomic changes in society over the last 900 years (Newton, 2011); however, some woodland elements of this system are currently undergoing major changes in structure and composition (Martin et al., 2015). Possible causes of dieback have been attributed to the co‐occurrence of multiple stressors, such as droughts and novel pathogenic fungi (Martin et al., 2015).

Our research focused on the broadleaved woodlands of the National Park, which are highly valued for their biodiversity, recreational opportunities and amenities (Newton, 2010), and managed with the dual purposes of (1) conserving and enhancing the natural beauty, wildlife, and cultural heritage and (2) promoting opportunities for the understanding and enjoyment of the special qualities of the Park by the public, as set out by the Environmental Act 1995. Management decision making plays a crucial part in meeting the dual statutory purposes. These woodlands were identified by selecting the SSSI management units with >50% of the basal area represented by broadleaved species and comprised 6,909 ha (Appendix S1). They include ancient pasture‐woodlands originating in the 18th century or earlier, shaped by the presence of grazing and traditional pollarding of trees (Peterken, Spencer, & Field, 1996), and “enclosed” woodlands that historically have been managed for timber production and have at times been protected by stock fences. In addition to their ability to provide timber, today enclosed woodlands are increasingly recognized for their nature conservation and recreation value (Forestry Commission 2008). The tree biomass is dominated by *Quercus robur* (47%) and *Fagus sylvatica* (33%), with an understorey of *Ilex aquifolium* (9%) and an admixture of *Betula pendula* (4.5%), *Crataegus monogyna* (1.3%), and *Taxus baccata* (0.9%). Details of the species characteristics found in the broadleaved woodlands were based on Newton et al. (2013) (Table 1). The main soil types are surface water gleys (84%), ground water gleys (9%), brown earths (7%), and podzols (0.2%) based on the National Soil Resources Institute (2007) and Pyatt et al. (2003). The local climate is temperate oceanic with a mean (±*SD*) annual precipitation of 832 ± 150 mm and mean (±*SD*) annual temperature of 10.17 ± 0.64°C between 1957 and 2014 (Met Office 2015).

**Table 1:** Details of the species characteristics encountered in the broadleaved woodlands of the New Forest National Park

*00*	Long	Mat	ShT	FiT	EffSD	MaxSD	VRP	Min VRP	Max VRP	P‐FiR
*Acer campestre*	200	10	3	1	80	120	1	10	120	None
*Acer pseudoplatanus*	150	12	4	1	120	400	1	10	100	None
*Alnus glutinosa*	250	12	3	1	120	200	1	10	200	None
*Betula pendula*	160	18	2	1	200	1,600	1	10	120	None
*Carpinus betulus*	250	20	4	1	90	130	1	10	150	None
*Castanea sativa*	300	35	3	1	300	700	1	10	250	None
*Corylus avellana*	80	10	4	1	300	700	1	10	80	None
*Crataegus monogyna*	150	4	2	1	300	700	1	10	100	None
*Fagus sylvatica*	500	55	5	1	300	700	1	10	300	None
*Frangula alnus*	80	3	2	2	300	700	1	10	30	None
*Fraxinus excelsior*	200	17	3	1	90	120	1	10	200	None
*Ilex aquifolium*	300	10	3	1	300	700	1	10	300	None
*Malus sylvestris*	130	8	2	1	300	700	1	10	100	None
*Picea abies*	300	40	2	1	100	120	0	0	0	None
*Picea sitchensis*	300	22	2	1	100	120	0	0	0	None
*Pinus nigra*	350	22	2	1	100	150	0	0	0	None
*Pinus sylvestris*	300	12	2	1	100	1,000	0	0	0	None
*Populus alba*	250	7	2	1	500	1,600	1	10	250	None
*Prunus spinosa*	60	4	2	1	300	700	1	10	60	None
*Pseudotsuga menziesii*	400	12	2	3	120	380	0	0	0	None
*Quercus robur*	500	60	2	1	300	700	1	10	400	None
*Quercus rubra*	200	22	4	3	300	700	0	0	0	None
*Salix cinerea*	90	35	2	1	1,000	1,600	1	10	70	None
*Sorbus aria*	150	6	2	1	300	700	0	0	0	None
*Sorbus aucuparia*	100	15	2	1	300	700	1	10	100	None
*Sorbus torminalis*	100	13	4	1	300	700	1	10	100	None
*Taxus baccata*	3,000	20	4	1	300	700	0	0	0	None
*Tsuga heterophylla*	400	15	2	1	120	160	0	0	0	None
*Viburnum opulus*	50	5	2	1	300	700	1	10	40	None

Long, longevity (years); Mat, age of sexual maturity (years); ShT, shade tolerance (1–5); FiT, fire tolerance (1–5); EffSD, effective seed dispersal distance (m); MaxSD, maximum seed dispersal distance (m); VRP, vegetative reproduction probability (0–1); MinVRP, minimum age of vegetative reproduction (years); MaxVRP, maximum age of vegetative reproduction (years); P‐FiR, postfire regeneration form (none, resprouting, or serotiny). Values were based on Newton et al. (2013).

### 2.2 Study design

Two main sets of scenarios were developed to explore the impact of increasing disturbance on the provisioning of ecosystem services and biodiversity: “pulse” and “pulse+press” sets. Under the pulse set, forest dynamics following a one‐off disturbance, such as a windthrow event or pathogen attack, were explored using an increasing percentage area to which the disturbance was applied (0%, 20%, 40%, 60%, 80%, and 100%). The disturbance commenced after 5 years of the simulation and lasted for 1 year, randomly removing the dominant tree species with > 10 cm diameter at breast height (dbh). Under the pulse + press set, the six disturbance intensities explored during the pulse set were followed by a continuous disturbance, simulating the current levels of browsing of trees by livestock and deer (Newton et al., 2013). In total, 12 scenarios were simulated and each scenario was replicated three times, owing to the stochastic nature of disturbance events. Due to the low variation between the three replicates for each scenario, AGB of each replicate was less than ±5% of the mean of replicates at the end of the simulation. Scenarios were developed over a time frame relevant to decision making (100 years; Forestry Commission 2016) and were run at a 50‐m resolution (or cell size).

All scenarios were simulated using a spatially dynamic model (LANDIS‐II v.6.0; Scheller et al., 2007), designed to simulate the spatiotemporal dynamics of forested landscapes through the incorporation of a number of ecological processes including succession, disturbances, and seed dispersal (Scheller et al., 2007). Following guidance for application of the model (Scheller & Lucash, 2014), the landscape was divided into 25 ecoregions on the basis of elevation and soil type, based on Newton et al. (2013). Mortality events were modeled using the harvesting succession extension (Base Harvest v2.2). Tree establishment, forest succession, and C and N dynamics were modeled using the Century Succession Extension (v4.0; Scheller et al., 2012), which is derived from the original CENTURY soil model (Parton, Anderson, Cole, & Stewart, 1983). The model was parameterized and calibrated using empirical data from the site and the scientific literature. Procedures for gathering the model inputs and model calibration are described in Appendix S2.

**Figure 1:**
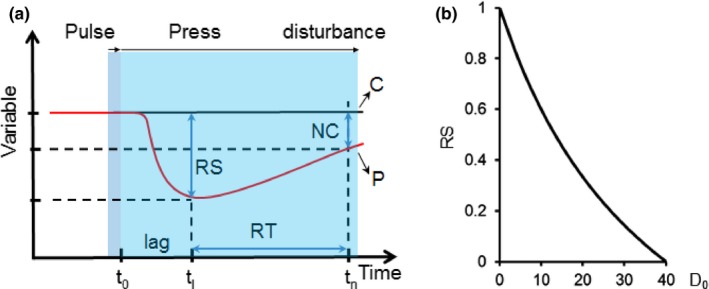
(a) Example of the resistance (RS), recovery time (RT), and net change (NC) of a response variable to a pulse and press disturbance. The black upper line represents the control variable (C) and the red line represents the perturbed variable (P); (b) Changes in RS with changes in *D*_0_ (i.e., *C*_0_–*P*_l_), when *C*_0_ is fixed at 40. Adapted from Shade et al. (2012) and Orwin and Wardle (2004)

Three properties of resilience were calculated, following Nimmo et al. (2015): resistance, recovery time, and net change. Resistance was measured as the magnitude of change of each variable (i.e., ecosystem service or biodiversity measure) caused by the disturbance, using the index proposed by Orwin and Wardle (2004):

(1) $$\text{resistance}\;\left(t_{l}\right)=1-\frac{2D_{0}}{C_{0}+D_{0}}$$

where *D*_0_ is the difference between the control variable (C) at time *t*_0_, and the disturbed variable (P) after a one‐off disturbance has occurred (i.e., end of year 5; Figure 1a). The index was bounded by 0 and +1, with a value of +1 showing maximum resistance and lower values showing less resistance. In those cases where *D*_0_ > *C*_0_ (indicating an increase in the value of the variable), the index was set to 1 to avoid the index giving a negative value of resistance (Figure 1b). Recovery time was measured as the time taken for each variable to return to the predisturbance value (Pimm, 1984). Maximum recovery time was 100 years to coincide with the time frame of the scenarios, relevant to decision making (Forestry Commission 2016). In those cases where resistance (*t*_l_) was set to 1, recovery time was considered nonapplicable and therefore was not calculated. It is worth noting that recovery time is referred to as “resilience” by Grimm and Wissel (1997) and Donohue et al. (2016). Systems with shorter recovery times are more resilient than those with longer recovery time (Donohue et al., 2016). Net change was measured by comparing each variable at the end of the simulation with the predisturbance value, following Nimmo et al. (2015).

Linear mixed models (LMMs) were fitted to estimate the relationships between resistance, recovery time, and net change and the degree of disturbance for each ecosystem service and biodiversity measure. To improve models performance and interpretability of coefficients, the degree of disturbance was standardized prior to analysis using the methods in Schielzeth (2010). Models fitted included null (*M* = *B*_0_ + Re), linear (*M* = B_0_ + *D B*_1_ + Re), and quadratic terms (*M* = B_0_ + *D B*_1_ + *D B*_2_^2^ + Re), with scenario replicates as a random effect (where *M* is the metric of interest, *B*_0_ is the model intercept, *D* is the degree of disturbance, *B*_1_ and *B*_2_ are parameters relating to the slope, and Re is a random effect that identifies the different model replicates). Model selection was performed by comparing models AICc, with the best model having the lowest AICc. The coefficients and the nature of the best models were recorded along with *R* ^2^ values following the methods of Nakagawa and Schielzeth (2013). A variable was considered to show a threshold if the best model included a quadratic term, indicating a nonlinear relationship, and its marginal *R* ^2^ value was >0.9. These criteria were based on what we considered to be good practice, but it should be noted that use of different criteria might have yielded different results. A paired Wilcoxon signed rank test was used to test differences between the pulse and the pulse + press values of resistance, recovery time, and net change. Spearman correlation analyses between resistance, recovery time, and net change were performed for each of the variables. All analyses were conducted in R 3.2.2. (R Core Team, 2015), using the lme4 package (Bates, Mächler, Bolker, & Walker, 2014) for mixed models, and the qdapTools (Goodrich, Kurkiewicz, Muller, & Rinker, 2015) for correlations.

### 2.3 Ecosystem services and biodiversity data sets

**Figure 2:**
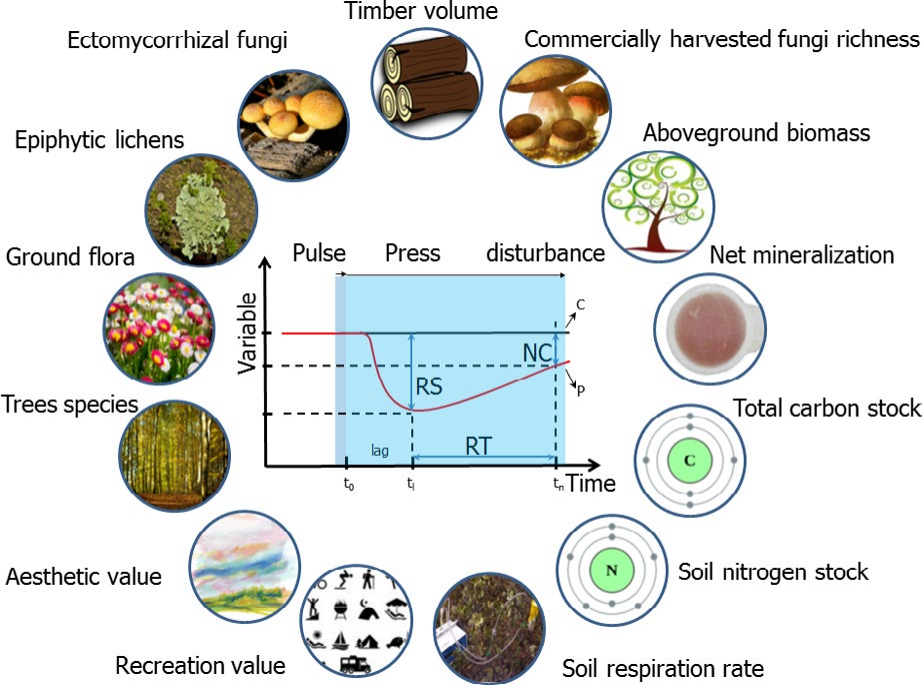
Diagram synthesizing the 13 variables selected (outside circles) and the study design employed to measure their resilience (inside graph). For explanation of graph labels, see Figure 1. For full description of the study design, see text

Nine ecosystem services and four biodiversity measures were selected, based on their importance in forest ecosystems: aboveground biomass (Mg/ha), aesthetic value, commercially harvested fungi richness, net nitrogen (N) mineralization absorbed to ionic resins [(μg NO_3_^−^ + NH_4_^+^)/capsule)], recreation value, soil nitrogen stock (Mg N/ha), soil respiration rate (μmols m^2^/s), timber volume (m^3^/ha), total carbon stock (Mg C/ha), and species richness of ectomycorrhizal fungi (ECM), ground flora, epiphytic lichens, and trees (Figure 2).

Aboveground biomass, total C stock, and soil N stock were calculated from the Century Extension of LANDIS‐II. Timber volume was calculated by multiplying the aboveground biomass of the species important for timber production (i.e., *Quercus robur* and *Fagus sylvatica*) for their respective nominal specific gravity (Jenkins et al., 2011).

Net N mineralization, soil respiration rate, species richness of commercially harvested and ectomycorrhizal fungi, ground flora, and epiphytic lichens were measured in the field along twelve replicate gradients of temperate forest dieback, from intact forest to grassland. Recreation and aesthetic values were measured by conducting a questionnaire survey of 200 visitors distributed equally across ten car parks within the SSSI New Forest boundary (see Appendix S3). For each of these variables, LMMs were fitted to estimate the relationships between aboveground biomass (AGB) and the variables. A value for each of the 50‐m cells associated with broadleaved woodlands was then derived from the model‐averaged coefficients of the LMMs fitted. Recreation and aesthetic values, which were assessed on a score of 1‐5, were transformed to proportions by dividing all values by 5 and performing a logit transform, based on Warton and Hui (2011). For variables of species richness, a Poisson error structure was used, while Gaussian errors were used for all other variables. Further information about the LMMs fitted is presented in Appendix S4.

## 3 RESULTS

### 3.1 Ecosystem services and biodiversity spatial and temporal variation

**Figure 3:**
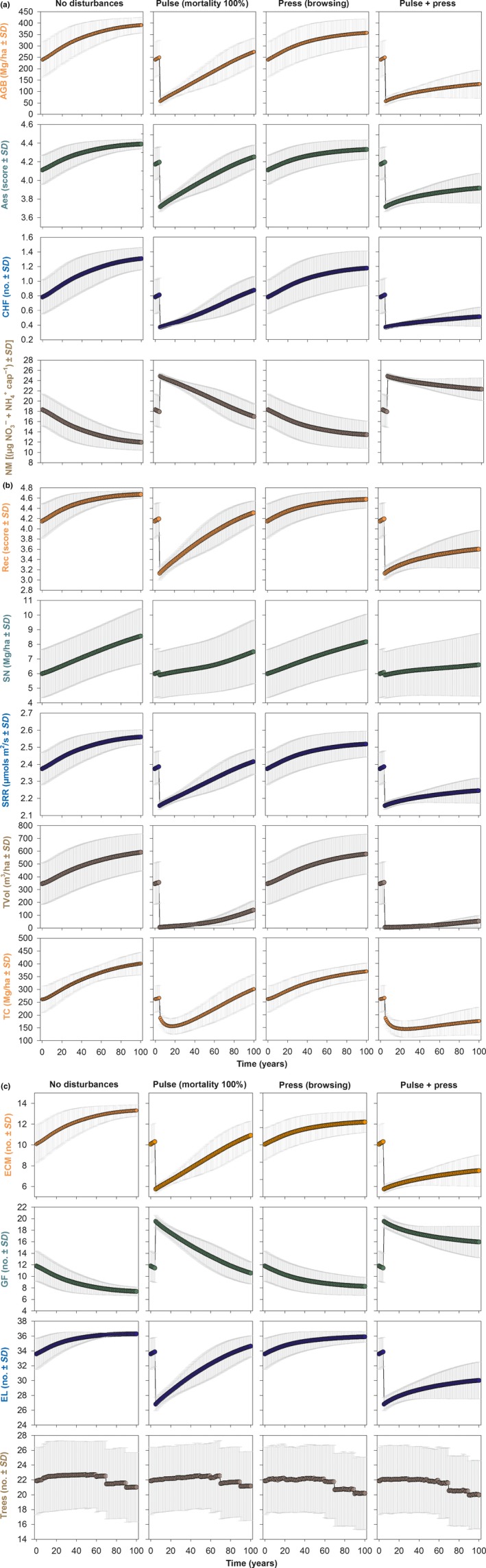
Ecosystem services and biodiversity measures of different degrees of disturbance simulated by pulse and pulse+press set scenarios over a 100‐year time span. Values represent landscape level means weighted by ecoregions (in color) and standard deviations (in gray) across three replicates. (a) Aes, aesthetic value; CHF, commercially harvested fungi richness; NM net N mineralization; (b) Rec, recreation value; SN, soil nitrogen stock; SRR, soil respiration rate; TVol, timber volume; TC, total carbon stock; (c) ECM, ectomycorrhizal fungi richness; GF, ground flora richness; EL, epiphytic lichen richness; and Trees, tree species richness. Note that for illustrative purposes, only four of the 12 scenarios are presented here. The first two columns illustrate the least and the most severe of the pulse set scenarios (0% and 100% pulse disturbance), whereas the last two columns illustrate the least and the most severe of the pulse + press scenarios (press only and 100% pulse disturbance combined with press). See text for a full description of the scenarios

All of the ecosystem services and biodiversity measures studied varied spatially between ecoregions. Most variables (10/13) demonstrated a sudden decrease after the pulse disturbance was applied and started to increase back to a predisturbance value with time. Conversely, net N mineralization and ground flora richness displayed an increase after the pulse disturbance was applied, whereas tree species richness demonstrated little change with time (Figure 3).

### 3.2 Resistance

**Figure 4:**
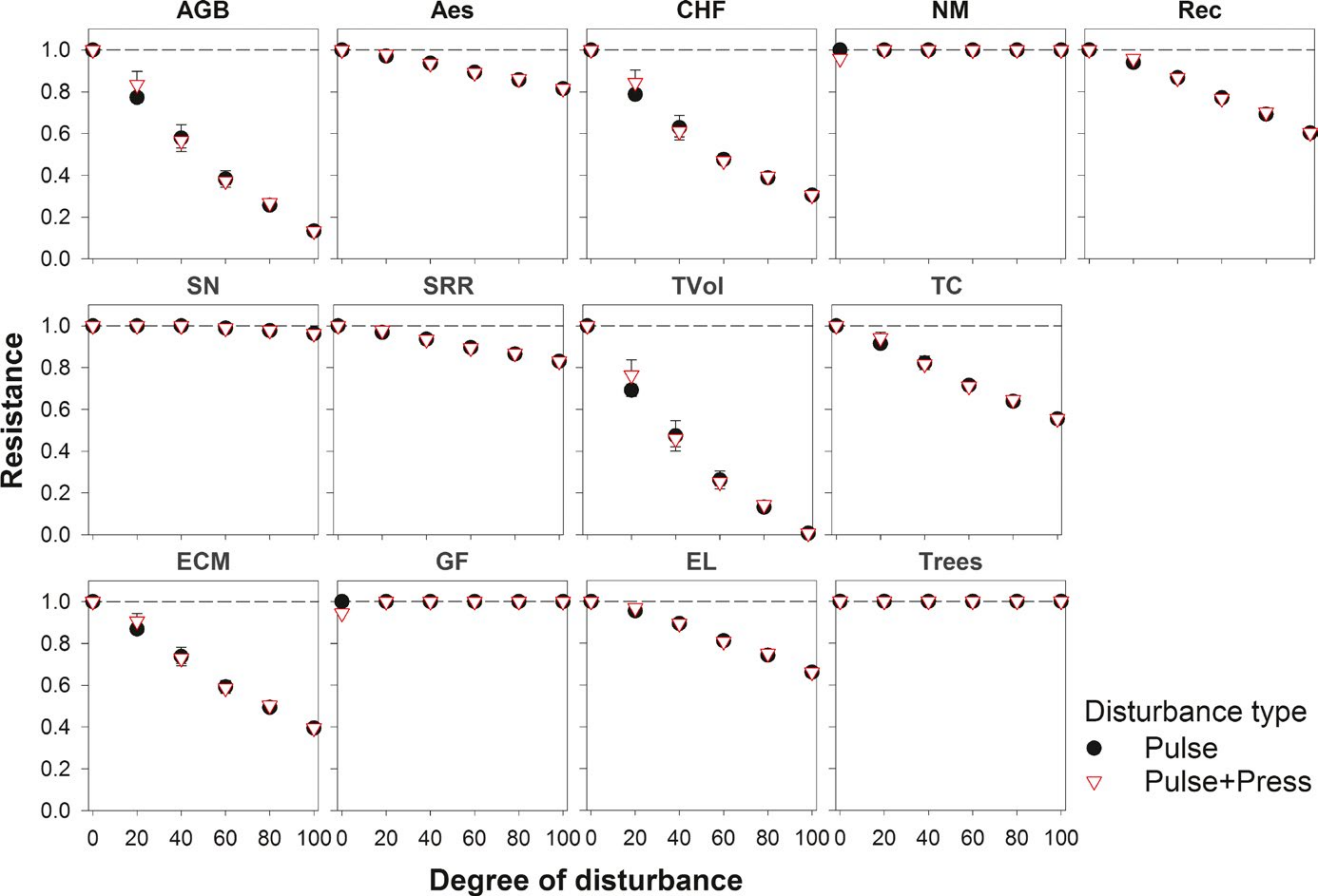
Ecosystem services and biodiversity resistance along an increasing degree of disturbance simulated by pulse and pulse–press set scenarios over 100 years. Values represent landscape level means and standard deviations across three replicates. See Section 3.3 for more details. AGB, aboveground biomass; Aes, aesthetic value; CHF, commercially harvested fungi richness; NM net N mineralization; Rec, recreation value; SN, soil nitrogen stock; SRR, soil respiration rate; TVol, timber volume; TC, total carbon stock; ECM, ectomycorrhizal fungi richness; GF, ground flora richness; EL, epiphytic lichen richness; Trees, tree species richness

The majority of the ecosystem services studied (8/9) showed a linear decline in resistance with increasing disturbance intensity. Only net N mineralization rate was resistant to disturbance. Timber volume and AGB demonstrated steeper declines, whereas declines in soil nitrogen stock and respiration rate were less rapid. Half of the biodiversity measures (2/4) showed a linear decline in resistance with increasing disturbance intensity, while the other half were resistant over time. Overall, resistance measures did not differ between pulse and pulse + press sets of scenarios (Figure 4, Appendix S5).

### 3.3 Recovery

**Figure 5:**
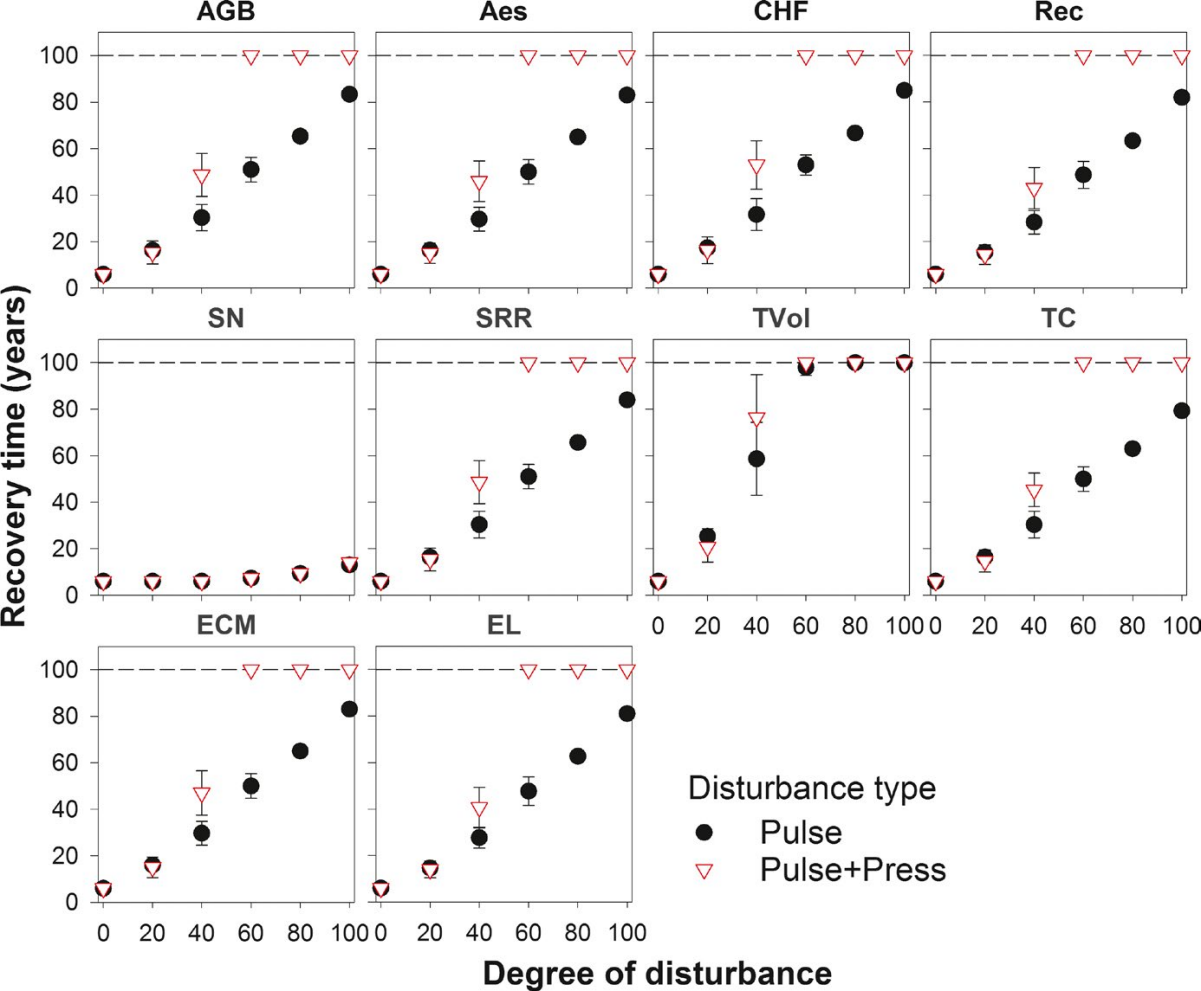
Ecosystem services and biodiversity recovery time along an increasing degree of disturbance simulated by pulse and pulse + press set scenarios over 100 years. Values represent landscape level means and standard deviations across three replicates. Note that values on the reference line indicate a recovery time >100 years. Recovery time for NM, GF, and Trees is omitted as not applicable. See Section 3.3 for more details. For explanation of plot labels, see Figure 4

All of the ecosystem service and biodiversity measures showed an increase in recovery time with increasing disturbance intensity. Recovery time increased relatively rapidly for timber volume and richness of commercially harvested fungi, and relatively slowly for epiphytic lichens richness and recreation value. Recovery time for soil nitrogen stock and timber volume did not differ between pulse and pulse + press scenarios. For all of the other ecosystem service and biodiversity measures, values diverged between the two sets of scenarios after disturbance was applied to >20% of the landscape. In all but one case, ecosystem service and biodiversity measures did not recover to predisturbance values at high disturbance levels >60%), even after 100 years (Figure 5; Appendix S5).

### 3.4 Net change

**Figure 6:**
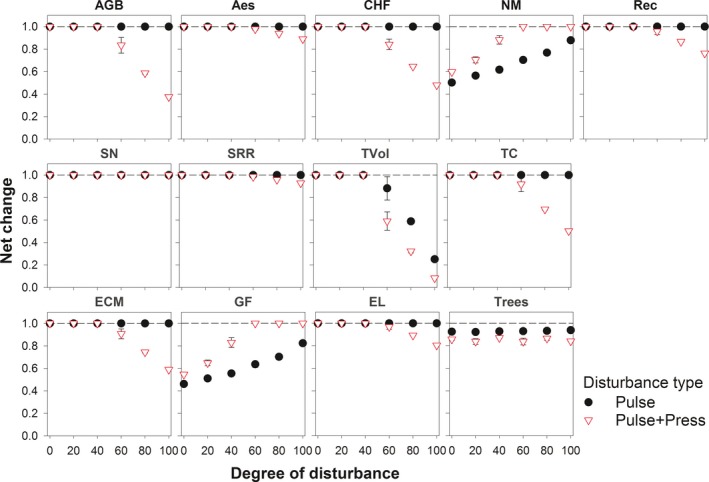
Ecosystem services and biodiversity net change along an increasing degree of disturbance simulated by pulse and pulse + press set scenarios over a 100‐year time span. Values represent landscape level means and standard deviations across three replicates. See Section 3.3 for more details. For explanation of plot labels, see Figure 4

Under the pulse set, the majority of the ecosystem services and biodiversity measures (10/13) demonstrated an ability to recover to predisturbance values after 100 years. Ground flora richness and net N mineralization showed an increase in net change with disturbance intensity, whereas timber volume showed a decrease. Under the pulse+press set, net change exhibited a threshold response in the majority of the cases (11/13). In nine cases, net change started to decline sharply when disturbance was applied to >40% of the landscape, whereas in two cases net change showed a quadratic increase with disturbance intensity. Soil nitrogen stock and tree species richness were best modeled by null models. Overall, with the exception of soil nitrogen stock, all of the net change measures differed between pulse and pulse + press scenario sets (Figure 6; Appendix S5).

### 3.5 Relationship between resistance, recovery, and net change

Under the pulse scenarios, all of the nonresistant ecosystem services and biodiversity measures (10/13) demonstrated a negative correlation between resistance and recovery time. Only in the case of timber volume, resistance and recovery time were correlated with net change (positively and negatively, respectively). Similarly to the pulse scenarios, under the pulse+press scenarios, 10 of 13 of the measures demonstrated a negative correlation between resistance and recovery time. Resistance was also positively correlated with net change in 11 of 13 cases. Recovery time was negatively correlated with net change in nine of 13 cases (Appendices S5 and S6).

## 4 DISCUSSION

Despite the emergence of fostering resilience as a new paradigm of forest ecosystem management (Millar & Stephenson, 2015; Newton & Cantarello, 2015; Seidl et al., 2016), quantifiable metrics of resilience to changing disturbance regimes are severely lacking. Our study provides a quantitative assessment approach for forest ecosystems that focuses on three measurable elements of resilience, namely resistance, recovery, and net change, and explores the spatiotemporal patterns of different disturbance intensities. Our approach takes the “multidimensionality” of stability concepts into account, which has two dimensions: (1) several stability properties need to be assessed in parallel to provide a comprehensive understanding of the mechanisms underpinning a system (Donohue et al., 2016; Grimm & Wissel, 1997; Pimm, 1984); (2) there is a need to explore the response of systems to different types of disturbance, observed at different spatial and temporal scales, for several state variables and reference dynamics. This has been dubbed “ecological checklist” by Grimm and Wissel (1997). The most striking result of our study was the detection of thresholds in net change under a pulse + press scenario for the majority of the ecosystem services and biodiversity measures. In our specific case, net change started to decline sharply when disturbance affected >40% of the landscape. Thresholds in net change were not observed under the pulse scenarios, with the exception of timber volume and ground flora species richness. Thresholds were most pronounced for AGB and timber volume with respect to the ecosystem services, and ECM and ground flora species richness with respect to the biodiversity measures.

Threshold responses to environmental change are currently the focus of major scientific interest and societal concern (Mace, Hails, Cryle, Harlow, & Clarke, 2015; Oliver et al., 2015; Steffen et al., 2015), as when a threshold is crossed, a small perturbation may lead to major ecological change. However, the evidence for such thresholds in terrestrial ecosystems is currently limited. Our results indicate that if the temperate forest examined here continues to be subjected to the browsing intensity that it experiences at present, no major changes in ecosystem service provision and biodiversity are forecast (response type i of Millar & Stephenson, 2015). However, when browsing is combined with a pulse disturbance that causes tree mortality, such as a windthrow event or pathogen attack, the forest displays a threshold response. If pulse and press disturbances are applied to ≤40% of the area, the forest is still able to sustain primary ecosystem services (responses type 2), whereas when these disturbances affect >40% of the forest area, recovery and net change in both biodiversity and ecosystem services is significantly altered (response type 3).

The mechanisms underlying ecological thresholds are unclear, but imply the existence of positive feedbacks between variables influencing the system (Scheffer et al., 2012).The thresholds identified in this study may be attributable to positive feedbacks between the pulse and press disturbances. While the pulse disturbance reduces AGB, the press disturbance limits tree recruitment, which could accelerate a decline in future tree growth. Many wood‐pastures habitats in Europe suffer from regeneration failure, primarily because of high herbivore pressure (Bergmeier, Petermann, & Schröder, 2010). If the loss of large trees is not compensated by regeneration and browsing intensities remain high, the result will be conversion of woodland to open pastures, a process that we have documented in our study site in some locations (Martin et al., 2015). Results from long‐term monitoring data in our site showed that over a period of 50 years, basal area declined by 33% and juvenile tree densities were reduced by ~70% (Martin et al., 2015). The threshold in AGB observed here was less pronounced than in timber volume, as the species important for timber production coincided with the dominant species that were extirpated by the disturbances. As disturbance intensifies, relatively shade‐tolerant dominant tree species are replaced by pioneer species (Newton, 2010), allowing AGB to recover more quickly compared to timber volume. The threshold observed in ECM richness could be attributable to the decline in tree root density associated with the loss of AGB and supports the findings of Treu et al. (2014) who showed ECM richness declining along a gradient of tree mortality. ECM richness is found to be dependent on tree root density, leaf area, and a sufficient supply of carbohydrate from the tree host (Yarwood, Myrold, & Hogberg, 2009) and can decrease following tree harvesting and insect attacks (Teste, Lieffers, & Strelkov, 2012; Treu et al., 2014). In the presence of an ECM richness decline, tree species may suffer significant reduction in growth and regeneration, resulting in a positive feedback between ECM and AGB decline (Simard et al., 2012). The thresholds in ground flora richness can be explained by well‐known patterns of successional changes in forest ecosystems (Bormann & Likens, 1979). For example, Zenner, Kabrick, Jensen, Peck, and Grabner (2006) demonstrated that ground flora richness increased proportionally along a gradient of harvest intensity, in accordance with the results found in our study.

The current study presents a few issues that should be borne in mind when interpreting the results obtained. With regard to the ecosystem services and biodiversity measures explored, total carbon stock, soil nitrogen stock, and tree species richness were calculated from the Century Extension of LANDIS‐II, which common to all ecological models is subject to a number of limitations and assumptions (Appendix S2). AGB was used as indicator for the remaining of the ecosystem services and biodiversity measures. This was based on data collected during previous research undertaken by Ref. Evans et al. (in press) and Gosal (2016) who measured a range of ecosystem services and biodiversity metrics along a gradient of woodland dieback, using basal area as a measure of forest structure. Basal area and AGB are among the indicators commonly found to be significantly related to biodiversity and ecosystem services (Cantarello & Newton, 2008; Harrison et al., 2014). However, in our study, some of the ecosystem services (namely aesthetic and recreation value, net N mineralization, and soil respiration rate) showed a low marginal *R* ^2^ in the linear mixed models fitted (Appendix S4), and therefore, the results for these measures need to be considered with caution. Further research is required to examine the resilience of these measures.

Further, it should be noted that there are other issues to consider when interpreting the results obtained. Here, we quantified resilience through the three metrics of resistance, recovery, and net change. This represents an advance over adopting a one‐dimensional perspective to assessing resilience, which as noted by Donohue et al. (2016) has been a feature of many previous studies., However, other components of resilience could have been adopted, such as asymptotic stability, variability and persistence (Pimm, 1984), or robustness (Donohue et al., 2016).

Despite these limitations, our results have a number of implications for management. Current forest management in the UK is guided by national and regional forestry policies (Forestry Commission 2010; European Union, 2015), as well as specific management objectives for individual sites (Forestry Commission 2016). In sites such as the New Forest, in which many species are dependent on the maintenance of early successional communities, sustaining a disturbance regime can be critical to conserving biodiversity value (Newton, 2010). However, our results indicated that when current browsing intensities are combined with a pulse disturbance, such as a windthrow event or pathogen attack, thresholds effects can occur, leading to accelerated loss of ecosystem services and biodiversity. Today, there is increasing concern that many ecosystem services provided by forests in Europe will be affected negatively in coming decades, owing to the increased incidence of windthrow, bark beetle, and wildfires (Seidl, Schelhaas, Rammer, & Verkerk, 2014). Emerging diseases including acute oak decline, ask dieback, chestnut blight, Dutch elm disease, pine wilt, and Japanese larch disease are also causing increasing tree mortality in many parts of the world including the UK (Boyd, Freer‐Smith, Gilligan, & Godfray, 2013; Pautasso, Aas, Queloz, & Holdenrieder, 2013). The current results highlight how such different perturbations can potentially interact, leading to the loss of both biodiversity and ecosystem services.

Managers could potentially use measurements of resilience, and identification of thresholds of response to disturbance, to develop interventions specifically intended to increase forest resilience. In the case study examined here, specific recommendations to enhance resilience in the short–medium term could include: (1) protecting tree regeneration from high herbivore pressure, which limits recruitment of trees, and (2) limiting the current management practice of tree cutting and heathland burning outside the woodland units so that trees might colonize nearby grassland and heathland and adapt to the new environmental conditions. However, this would mean accepting woodlands collapse in some parts of the landscape and expand in other areas, which could result in potential negative impacts on biodiversity and ecosystem service provision at the landscape scale. For example, native ancient woodlands are highly valued for their biodiversity, and their loss could have implications for many species of conservation interest that depend on them (Bergmeier et al., 2010; Plieninger et al., 2015). Biodiversity loss also has the potential to alter ecosystem functioning (Duffy, 2009). Other recommendations to increase forest resilience have been proposed including planting resilient tree species that tolerate a variety of climates and the selection and use of clones resistant to pests and diseases (Fares, Mugnozza, Corona, & Palahi, 2015; Forestry Commission 2015). However, as noted by Newton (2016), these recommendations would undermine current efforts to halt biodiversity loss. For example, tree species diversification could endanger the exceptional biodiversity value of ancient native woodlands (Bruun, Heilmann‐Clausen, & Ejrnaes, 2015). Management practices that preserve natural ecosystem processes are likely to be more effective in supporting forest biodiversity and resilience (Jonsson, Pe'er, & Svoboda, 2015).

These results also have implications for how resilience is best measured. Here, following Grimm and Wissel (1997) and Nimmo et al. (2015), we assessed resilience as three independent components. However, we also examined whether these different components were correlated with each other, to test whether they could potentially be combined into a single measure or index of resilience. In most cases under the pulse + press scenarios, resistance and net change were positively correlated, whereas resistance and recovery were negatively correlated. However under the pulse scenarios, resistance and recovery were generally not correlated with net change. This indicates that these different components should be differentiated in analysis of resilience, as they may respond differently to disturbance. Consideration of all three components under a combined single measure of resilience could therefore obscure important ecological changes occurring in forests. Resilience is increasingly being incorporated into many environmental management policies at national and global scales, despite the current measurement difficulty, which increases the risk of its misuse (Newton, 2016). Such risks could potentially be addressed using the kinds of measurement approaches described here and by developing specific management responses to support each of the three resilience components individually.

## CONFLICT OF INTEREST

None declared.

## AUTHORS’ CONTRIBUTION

A.C.N. and E.C conceived and designed the study. P.M.E., E.C., and A.G collected field data. E.C parameterized, calibrated, and run model with contribution from M.S.L. E.C. and P.A.M analyzed the data. E.C. and A.C.N. wrote the manuscript with contributions from all authors.

## DATA ACCESSIBILITY

Raw data are in the process of being archived by The Environmental Information Data Centre (EIDC): http://eidc.ceh.ac.uk/

## References

[ece33491-bib-0001] Allen, C. R. , Angeler, D. G. , Cumming, G. S. , Folke, C. , Twidwell, D. , & Uden, D. R. (2016). Quantifying spatial resilience. Journal of Applied Ecology, 53, 625–635.

[ece33491-bib-0002] Allen, C. D. , Breshears, D. D. , & McDowell, N. G. (2015). On underestimation of global vulnerability to tree mortality and forest die‐off from hotter drought in the Anthropocene. Ecosphere, 6, 000–000.

[ece33491-bib-0003] Angeler, D. G. , Allen, C. R. , Birge, H. E. , Drakare, S. , McKie, B. G. , & Johnson, R. K. (2014). Assessing and managing freshwater ecosystems vulnerable to environmental change. Ambio, 43, 113–129. 25403974 10.1007/s13280-014-0566-z PMC4235931

[ece33491-bib-0004] Bates, D. , Mächler, M. , Bolker, B. M. , & Walker, S. C. (2014). Fitting linear mixed‐effects models using lme4. Madison, WI, USA: Department of Statistics, University of Wisconsin.

[ece33491-bib-0005] Bergmeier, E. , Petermann, J. , & Schröder, E. (2010). Geobotanical survey of wood‐pasture habitats in Europe: Diversity, threats and conservation. Biodiversity and Conservation, 19, 2995–3014.

[ece33491-bib-0006] Biggs, R. , Schluter, M. , Biggs, D. , Bohensky, E. L. , BurnSilver, S. , Cundill, G. , … West, P. C. (2012). Toward principles for enhancing the resilience of ecosystem services. Annual Review of Environment and Resources, 37(37), 421–448.

[ece33491-bib-0007] Bormann, F. H. , & Likens, G. E. (1979). Pattern and process in a forested ecosystem: Disturbance, development, and the steady state based on the Hubbard Brook ecosystem study. New York, NY, USA: Springer‐Verlag.

[ece33491-bib-0008] Boyd, I. L. , Freer‐Smith, P. H. , Gilligan, C. A. , & Godfray, H. C. J. (2013). The consequence of tree pests and diseases for ecosystem services. Science, 342, 823. 10.1126/science.1235773 24233727

[ece33491-bib-0009] Brand, F. S. , & Jax, K. (2007). Focusing the meaning(s) of resilience: Resilience as a descriptive concept and a boundary object. Ecology and Society, 12(1), 23 [online]. URL: http://www.ecologyandsociety.org/vol12/iss1/art23/.

[ece33491-bib-0010] Bruun, H. H. , Heilmann‐Clausen, J. , & Ejrnaes, R. (2015). Forests: See the trees and the wood. Nature, 521, 32–32. 10.1038/521032a 25951276

[ece33491-bib-0011] Cantarello, E. , Green, R. , & Westerhoff, D. (2010). The condition of The New Forest habitats: An overview In Newton A. C. (Ed.), Biodiversity in the new forest (pp. 124–131). Newbury, Berkshire: Pisces Publishing.

[ece33491-bib-0012] Cantarello, E. , & Newton, A. C. (2008). Identifying cost‐effective indicators to assess the conservation status of forested habitats in Natura 2000 sites. Forest Ecology and Management, 256, 815–826.

[ece33491-bib-0013] Collins, S. L. , Carpenter, S. R. , Swinton, S. M. , Orenstein, D. E. , Childers, D. L. , Gragson, T. L. , … Whitmer, A. C. (2011). An integrated conceptual framework for long‐term social–ecological research. Frontiers in Ecology and the Environment, 9, 351–357.

[ece33491-bib-0014] Dale, V. H. , Joyce, L. A. , McNulty, S. , Neilson, R. P. , Ayres, M. P. , Flannigan, M. D. , … Michael Wotton, B. (2001). Climate change and forest disturbances. BioScience, 51, 723–734.

[ece33491-bib-0015] Donohue, I. , Hillebrand, H. , Montoya, J. M. , Petchey, O. L. , Pimm, S. L. , Fowler, M. S. , … Yang, Q. (2016). Navigating the complexity of ecological stability. Ecology Letters, 19, 1172–1185. 27432641 10.1111/ele.12648

[ece33491-bib-0016] Duffy, J. E. (2009). Why biodiversity is important to the functioning of real‐world ecosystems. Frontiers in Ecology and the Environment, 7, 437–444.

[ece33491-bib-0017] Evans, P. M. , Newton, A. C. , Cantarello, E. , Martin, P. , Sanderson, N. , Jones, D. L. , Barsoum, N. , Cottrell, J. E. , A'Hara, S. W. , & Fuller, L. (2017). Thresholds of biodiversity and ecosystem function in a forest ecosystem undergoing dieback. Scientific Reports, 7, 6775. 28754979 10.1038/s41598-017-06082-6 PMC5533776

[ece33491-bib-0018] FAO (2012). State of the World's forests 2012. Rome, Italy: Food and Agriculture Organization of the United Nations (FAO).

[ece33491-bib-0019] Fares, S. , Mugnozza, G. S. , Corona, P. , & Palahi, M. (2015). Five steps for managing Europe's forests. Nature, 519, 407–409. 25810187 10.1038/519407a

[ece33491-bib-0020] Forestry Commission (2008). Crown lands management plan 2008–2013. Lyndhurst: Forestry Commission.

[ece33491-bib-0021] Forestry Commission (2010). Managing ancient and native woodland in England. Bristol, UK: Forestry Commission England.

[ece33491-bib-0022] Forestry Commission (2015) Adapting England's woodlands to be more resilient [Online]. UK, Forestry Commission Retrieved from: https://www.forestry.gov.uk/forestry/infd-8m6hdl#Ancient%20woodland. Accessed 18 May 2017.

[ece33491-bib-0023] Forestry Commission (2016). New forest inclosures: Forest design plans phase D. Lyndhurst: Forestry Commission.

[ece33491-bib-0024] Goodrich, B. , Kurkiewicz, D. , Muller, K. , & Rinker, T. (2015) Tools for the ‘qdap’ Package.

[ece33491-bib-0025] Gosal, A. (2016). A multifaceted approach to spatial analysis of ecosystem services: A case study in the New Forest National Park. Doctor of Philosophy, Bournemouth University.

[ece33491-bib-0026] Greenberg, C. H. , & Collins, B. S. (2015). Natural disturbances and historic range of variation: Type, frequency, severity, and post‐disturbance structure in central hardwood forests USA. Heidelberg, Germany: Springer International Publishing.

[ece33491-bib-0027] Grimm, V. , & Wissel, C. (1997). Babel, or the ecological stability discussions: An inventory and analysis of terminology and a guide for avoiding confusion. Oecologia, 109, 323–334. 28307528 10.1007/s004420050090

[ece33491-bib-0028] de Groot, R. , Brander, L. , van der Ploeg, S. , Costanza, R. , Bernard, F. , Braat, L. , … van Beukering, P. (2012). Global estimates of the value of ecosystems and their services in monetary units. Ecosystem Services, 1, 50–61.

[ece33491-bib-0029] Harrison, P. A. , Berry, P. M. , Simpson, G. , Haslett, J. R. , Blicharska, M. , Bucur, M. , … Turkelboom, F. (2014). Linkages between biodiversity attributes and ecosystem services: A systematic review. Ecosystem Services, 9, 191–203.

[ece33491-bib-0030] Hodgson, D. , McDonald, J. L. , & Hosken, D. J. (2015). What do you mean, ‘resilient’? Trends in Ecology & Evolution, 30, 503–506. 26159084 10.1016/j.tree.2015.06.010

[ece33491-bib-0031] Jenkins, T. A. R. , Mackie, E. D. , Matthews, R. W. , Miller, G. , Randle, T. J. , & White, M. E. (2011). FC woodland carbon code: Carbon assessment protocol. Edingburgh, UK: Forestry Commission.

[ece33491-bib-0032] Jonsson, B. G. , Pe'er, G. , & Svoboda, M. (2015). Forests: Not just timber plantations. Nature, 521, 32–32. 10.1038/521032b 25951273

[ece33491-bib-0033] Lindner, M. , Maroschek, M. , Netherer, S. , Kremer, A. , Barbati, A. , Garcia‐Gonzalo, J. , … Marchetti, M. (2010). Climate change impacts, adaptive capacity, and vulnerability of European forest ecosystems. Forest Ecology and Management, 259, 698–709.

[ece33491-bib-0034] Mace, G. M. , Hails, R. S. , Cryle, P. , Harlow, J. , & Clarke, S. J. (2015). REVIEW: Towards a risk register for natural capital. Journal of Applied Ecology, 52, 641–653. 27563153 10.1111/1365-2664.12431 PMC4979659

[ece33491-bib-0035] Martin, P. A. , Newton, A. C. , & Bullock, J. M. (2013). Carbon pools recover more quickly than plant biodiversity in tropical secondary forests. Proceedings of the Royal Society B: Biological Sciences, 280, 20132236 https://doi.org/10.1098/rspb.2013.2236 24197410 10.1098/rspb.2013.2236 PMC3826225

[ece33491-bib-0036] Martin, P. A. , Newton, A. C. , Cantarello, E. , & Evans, P. (2015). Stand dieback and collapse in a temperate forest and its impact on forest structure and biodiversity. Forest Ecology and Management, 358, 130–138.

[ece33491-bib-0037] MEA (2005). Ecosystems and human well‐being: Synthesis. Island Press, Washington, DC: Millennium Ecosystem Assessment (MEA).

[ece33491-bib-0038] Met Office (2015) UK climate ‐ Historic station data [Online]. Exeter, UK Available: http://www.metoffice.gov.uk/pub/data/weather/uk/climate/stationdata/hurndata.txt [Accessed 08/12/2016].

[ece33491-bib-0039] Millar, C. I. , & Stephenson, N. L. (2015). Temperate forest health in an era of emerging megadisturbance. Science, 349, 823–826. 26293954 10.1126/science.aaa9933

[ece33491-bib-0040] Nakagawa, S. , & Schielzeth, H. (2013). A general and simple method for obtaining *R* ^2^ from generalized linear mixed‐effects models. Methods in Ecology and Evolution, 4, 133–142.

[ece33491-bib-0041] Nash, K. L. , Graham, N. A. J. , Jennings, S. , Wilson, S. K. , & Bellwood, D. R. (2016). Herbivore cross‐scale redundancy supports response diversity and promotes coral reef resilience. Journal of Applied Ecology, 53, 646–655.

[ece33491-bib-0042] National Soil Resources Institute (2007). NATMAPvector, SOILSERIES agronomy and hydrology for the New Forest, Hampshire. Silsoe, Bedfordshire, UK: Cranfield University.

[ece33491-bib-0043] Nemec, K. T. , Chan, J. , Hoffman, C. , Spanbauer, T. L. , Hamm, J. A. , Allen, C. R. , Hefley, T. , Pan, D. , & Shrestha, P. (2013). Assessing resilience in stressed watersheds. Ecology and Society, 19(1), 34 https://doi.org/10.5751/ES-06156-190134

[ece33491-bib-0044] Newton A. C. (Ed.) (2010). Biodiversity in the New Forest. Newbury, Berkshire: Pisces Publishing.

[ece33491-bib-0045] Newton, A. C. (2011). Social‐ecological resilience and biodiversity conservation in a 900‐year‐old protected area. Ecology and Society, 16(4), 13 https://doi.org/10.5751/ES-04308-160413

[ece33491-bib-0046] Newton, A. C. (2016). Biodiversity risks of adopting resilience as a policy goal. Conservation Letters, 9, 369–376.

[ece33491-bib-0047] Newton, A. C. , & Cantarello, E. (2015). Restoration of forest resilience: An achievable goal? New Forests, 46, 645–668.

[ece33491-bib-0048] Newton, A. C. , Cantarello, E. , Tejedor, N. , & Myers, G. (2013). Dynamics and conservation management of a wooded landscape under high herbivore pressure. International Journal of Biodiversity, 2013, 15.

[ece33491-bib-0049] Nimmo, D. G. , Mac Nally, R. , Cunningham, S. C. , Haslem, A. , & Bennett, A. F. (2015). Vive la resistance: Reviving resistance for 21st century conservation. Trends in Ecology & Evolution, 30, 516–523. 26293697 10.1016/j.tree.2015.07.008

[ece33491-bib-0050] Oliver, T. H. , Heard, M. S. , Isaac, N. J. B. , Roy, D. B. , Procter, D. , Eigenbrod, F. , … Bullock, J. M. (2015). Biodiversity and resilience of ecosystem functions. Trends in Ecology & Evolution, 30, 673–684. 26437633 10.1016/j.tree.2015.08.009

[ece33491-bib-0051] Orwin, K. H. , & Wardle, D. A. (2004). New indices for quantifying the resistance and resilience of soil biota to exogenous disturbances. Soil Biology & Biochemistry, 36, 1907–1912.

[ece33491-bib-0052] Parton, W. J. , Anderson, D. W. , Cole, C. V. , & Stewart, J. W. B. (1983). Simulation of soil organic matter formation and mineralization in semiarid agroecosystems In Lowrance R. R., Todd R. L., Asmussen L. E., & Leonard R. A. (Eds.), Nutrient cycling in agricultural ecosystems (pp. 533–550). Athens, Georgia: College of Agriculture Experiment Stations, The University of Georgia.

[ece33491-bib-0053] Pautasso, M. , Aas, G. , Queloz, V. , & Holdenrieder, O. (2013). European ash (*Fraxinus excelsior*) dieback—A conservation biology challenge. Biological Conservation, 158, 37–49.

[ece33491-bib-0054] Peterken, G. , Spencer, J. , & Field, A. (1996). Maintaining the ancient & ornamental woodlands of the New Forest. Lyndhurst: Forestry Commission.

[ece33491-bib-0055] Pimm, S. L. (1984). The complexity and stability of ecosystems. Nature, 307, 321–326.

[ece33491-bib-0056] Plieninger, T. , Hartel, T. , Martín‐López, B. , Beaufoy, G. , Bergmeier, E. , Kirby, K. , … Van Uytvanck, J. (2015). Wood‐pastures of Europe: Geographic coverage, social–ecological values, conservation management, and policy implications. Biological Conservation, 190, 70–79.

[ece33491-bib-0057] Pyatt, G. , Spencer, J. , Hutchby, L. , Davani, S. , Flethcher, J. , & Purdy, K. (2003). Applying the ecological site classification in the Lowlands. *Technical paper 33* Edinburgh: Forestry Commission.

[ece33491-bib-0058] R Core Team (2015). R: A language and environment for statistical computing. Vienna, Austria: R Foundation for Statistical Computing.

[ece33491-bib-0059] Reyer, C. P. O. , Brouwers, N. , Rammig, A. , Brook, B. W. , Epila, J. , Grant, R. F. , … Villela, D. M. (2015). Forest resilience and tipping points at different spatio‐temporal scales: Approaches and challenges. Journal of Ecology, 103, 5–15.

[ece33491-bib-0060] Scheffer, M. , Carpenter, S. R. , Lenton, T. M. , Bascompte, J. , Brock, W. , Dakos, V. , … Vandermeer, J. (2012). Anticipating critical transitions. Science, 338, 344–348. 23087241 10.1126/science.1225244

[ece33491-bib-0061] Scheller, R. M. , Domingo, J. B. , Sturtevant, B. R. , Williams, J. S. , Rudy, A. , Gustafson, E. J. , & Mladenoff, D. J. (2007). Design, development, and application of LANDIS‐II, a spatial landscape simulation model with flexible temporal and spatial resolution. Ecological Modelling, 201, 409–419.

[ece33491-bib-0062] Scheller, R. M. , Kretchun, A. M. , Van Tuyl, S. , Clark, K. L. , Lucash, M. S. , & Hom, J. (2012). Divergent carbon dynamics under climate change in forests with diverse soils, tree species, and land use histories. Ecosphere, 3, 110.

[ece33491-bib-0063] Scheller, R. M. , & Lucash, M. S. (2014). Forecasting forested landscapes: An introduction to LANDIS‐II with exercises. North Charleston, SC, USA: CreateSpace Independent Publishing Platform.

[ece33491-bib-0064] Schielzeth, H. (2010). Simple means to improve the interpretability of regression coefficients. Methods in Ecology and Evolution, 1, 103–113.

[ece33491-bib-0065] Seidl, R. (2014). The shape of ecosystem management to come: anticipating risks and fostering resilience. BioScience, 64, 1159–1169. 25729079 10.1093/biosci/biu172 PMC4340566

[ece33491-bib-0066] Seidl, R. , Fernandes, P. M. , Fonseca, T. F. , Gillet, F. , Jönsson, A. M. , Merganičová, K. , … Mohren, F. (2011). Modelling natural disturbances in forest ecosystems: A review. Ecological Modelling, 222, 903–924.

[ece33491-bib-0067] Seidl, R. , Schelhaas, M.‐J. , Rammer, W. , & Verkerk, P. J. (2014). Increasing forest disturbances in Europe and their impact on carbon storage. Nature Climate Change, 4, 806–810. 10.1038/nclimate2318 PMC4340567 25737744

[ece33491-bib-0068] Seidl, R. , Spies, T. A. , Peterson, D. L. , Stephens, S. L. , & Hicke, J. A. (2016). Searching for resilience: Addressing the impacts of changing disturbance regimes on forest ecosystem services. Journal of Applied Ecology, 53, 120–129. 26966320 10.1111/1365-2664.12511 PMC4780065

[ece33491-bib-0069] Simard, S. W. , Beiler, K. J. , Bingham, M. A. , Deslippe, J. R. , Philip, L. J. , & Teste, F. P. (2012). Mycorrhizal networks: Mechanisms, ecology and modelling. Fungal Biology Reviews, 26, 39–60.

[ece33491-bib-0070] Spake, R. , Ezard, T. H. G. , Martin, P. A. , Newton, A. C. , & Doncaster, C. P. (2015). A meta‐analysis of functional group responses to forest recovery outside of the tropics. Conservation Biology, 29, 1695–1703. 26040756 10.1111/cobi.12548 PMC4973697

[ece33491-bib-0071] Standish, R. J. , Hobbs, R. J. , Mayfield, M. M. , Bestelmeyer, B. T. , Suding, K. N. , Battaglia, L. L. , … Thomas, P. A. (2014). Resilience in ecology: Abstraction, distraction, or where the action is? Biological Conservation, 177, 43–51.

[ece33491-bib-0072] Steffen, W. , Richardson, K. , Rockstrom, J. , Cornell, S. E. , Fetzer, I. , Bennett, E. M. , … Sorlin, S. (2015). Planetary boundaries: Guiding human development on a changing planet. Science, 347, 1259855 https://doi.org/10.1126/science.1259855 25592418 10.1126/science.1259855

[ece33491-bib-0073] Teste, F. P. , Lieffers, V. J. , & Strelkov, S. E. (2012). Ectomycorrhizal community responses to intensive forest management: Thinning alters impacts of fertilization. Plant and Soil, 360, 333–347.

[ece33491-bib-0074] Treu, R. , Karst, J. , Randall, M. , Pec, G. J. , Cigan, P. W. , Simard, S. W. , … Cahill Jr., J. F. (2014). Decline of ectomycorrhizal fungi following a mountain pine beetle epidemic. Ecology, 95, 1096–1103. 24933827 10.1890/13-1233.1

[ece33491-bib-0075] Trumbore, S. , Brando, P. , & Hartmann, H. (2015). Forest health and global change. Science, 349, 814–818. 26293952 10.1126/science.aac6759

[ece33491-bib-0076] Union, European (2015). Natura 2000 and forests. Luxembourg: Office for Official Publications of the European Communities.

[ece33491-bib-0077] Usbeck, T. , Wohlgemuth, T. , Dobbertin, M. , Pfister, C. , Burgi, A. , & Rebetez, M. (2010). Increasing storm damage to forests in Switzerland from 1858 to 2007. Agricultural and Forest Meteorology, 150, 47–55.

[ece33491-bib-0078] Walker, L. R. (1999). Ecosystems of disturbed ground. Amsterdam, The Netherlands: Elsevier Science.

[ece33491-bib-0079] Warton, D. I. , & Hui, F. K. C. (2011). The arcsine is asinine: The analysis of proportions in ecology. Ecology, 92, 3–10. 21560670 10.1890/10-0340.1

[ece33491-bib-0080] Weed, A. S. , Ayres, M. P. , & Hicke, J. A. (2013). Consequences of climate change for biotic disturbances in North American forests. Ecological Monographs, 83, 441–470.

[ece33491-bib-0081] Yarwood, S. A. , Myrold, D. D. , & Hogberg, M. N. (2009). Termination of belowground C allocation by trees alters soil fungal and bacterial communities in a boreal forest. FEMS Microbiology Ecology, 70, 151–162. 19656196 10.1111/j.1574-6941.2009.00733.x

[ece33491-bib-0082] Zenner, E. K. , Kabrick, J. M. , Jensen, R. G. , Peck, J. E. , & Grabner, J. K. (2006). Responses of ground flora to a gradient of harvest intensity in the Missouri Ozarks. Forest Ecology and Management, 222, 326–334.

