# Supplementary material for: Quantifying resilience of multiple ecosystem services and biodiversity in a temperate forest landscape
Source: Ecol Evol. 2017 Oct 16;7(22):9661–75. doi: 10.1002/ece3.3491 (PMC5696413; doi:10.1002/ece3.3491)
Supplement: Supplementary file 1 [file ECE3-7-9661-s001.docx]

## Supporting information

### **Appendix S1.** Distribution of the broadleaved woodlands within the New Forest SSI and National Park.
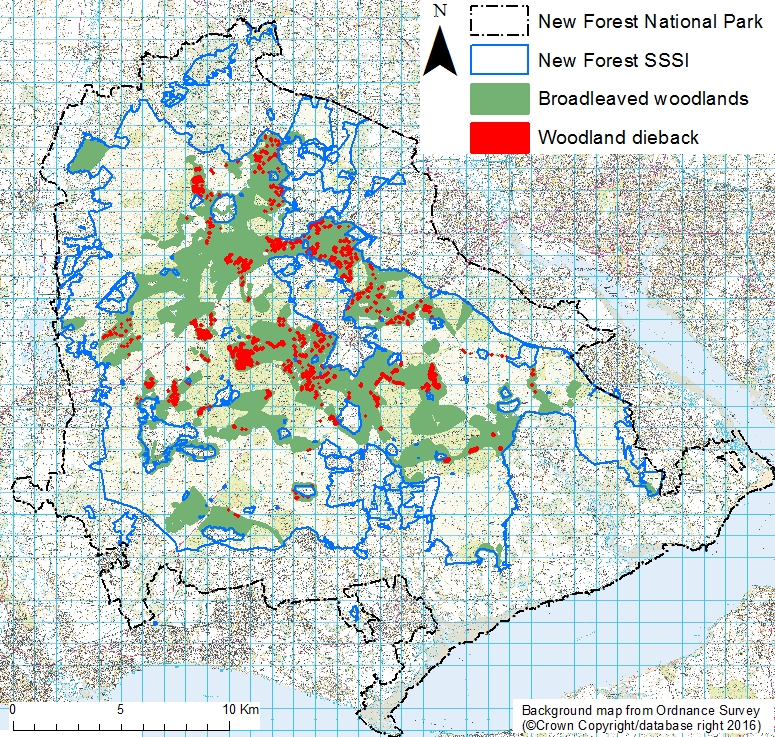


### **Appendix S2.** Description of the LANDIS-II model used to simulate the pulse+press sets scenarios.

LANDIS-II has been widely tested and applied extensively throughout the US ([Scheller *et al.* 2012](#_ENREF_2_36); [Lucash *et al.* 2014](#_ENREF_2_19); [Wang *et al.* 2014](#_ENREF_2_40); [Duveneck & Scheller 2015](#_ENREF_2_7); [Gustafson *et al.* 2015](#_ENREF_2_11)) and elsewhere ([Di Febbraro *et al.* 2015](#_ENREF_2_6); [Newton & Cantarello 2015](#_ENREF_2_25)). The model was originally developed to examine the interactions between succession and fire ([He & Mladenoff 1999](#_ENREF_2_12)). Over time the number of ecological processes has expanded to address both forest management and habitat conservation. Recent research has emphasized the impacts of different management actions on the successional dynamics ([Duveneck *et al.* 2014](#_ENREF_2_8)), wildlife habitat ([Pauli *et al.* 2015](#_ENREF_2_29)), the impact of land-use change on ecosystem services provision ([Blumstein & Thompson 2015](#_ENREF_2_1)), and the effects of disturbances and climate change on carbon and nitrogen cycling ([Kretchun *et al.* 2014](#_ENREF_2_16); [Lucash *et al.* 2014](#_ENREF_2_19)).

*Harvesting succession extension*

In our study we built on a previously parameterisation of LANDIS-II applied to the SSSI New Forest boundary, operating on 50 m cell raster maps ([Newton *et al.* 2013](#_ENREF_2_26)). Mortality events under the pulse and the pulse+press sets scenarios were modelled by using the LANDIS-II Harvesting succession extension (Base Harvest v2.2). Pulse disturbances were simulated by removing both *Quercus robur* (oak) and *Fagus sylvatica* (beech) with > 10 cm dbh. The press disturbance was based on a previous parameterisation of the harvesting extension, simulating the current levels of browsing of ponies and deer in the SSSI New Forest boundary ([Newton *et al.* 2013](#_ENREF_2_26)). In total 12 scenarios were simulated, 6 using an increasing percentage area to which the pulse disturbance was applied (0%, 20%, 40%, 60%, 80%, and 100%), and 6 where the six disturbance intensities explored during the pulse set were followed by the press disturbance. The target percentage area to be affected by the pulse disturbance was calculated iteratively, so that for each of the scenarios, a corresponding percentage of cells and age cohorts were removed (± 1%).

*Century Succession Extension*

Tree establishment, forest succession and C and N dynamics were modelled using the LANDIS-II Century Succession Extension (v4.0). This extension (hereon called ‘CENTURY’) simulates the internal cycling of C and N through the vegetation, detritus and soil. C and N pool flows and interactions with climate are described elsewhere ([Scheller *et al.* 2011b](#_ENREF_2_35); [Lucash *et al.* 2014](#_ENREF_2_19)). CENTURY also simulates above- and below-ground net primary productivity (NPP), net ecosystem exchange (NEE) and heterotrophic respiration (Rh) at a monthly time step. The extension requires three general input types: species-specific, functional groups and ecoregions inputs. Here we outline our procedures for gathering these inputs.

Initial species composition and age distribution for the simulations was based on [Newton *et al.* (2013)](#_ENREF_2_26). Species-specific inputs included 17 parameters which were estimated based on the life history presented in the literature ([Burns & Honkala 1990](#_ENREF_2_2); [Escudero *et al.* 1992](#_ENREF_2_9); [Sjöström 1993](#_ENREF_2_38); [Reich *et al.* 1996](#_ENREF_2_33); [Mediavilla & Escudero 2003](#_ENREF_2_20); [Pyatt *et al.* 2003](#_ENREF_2_32); [Hill *et al.* 2004](#_ENREF_2_13); [Ishii *et al.* 2009](#_ENREF_2_14); [Wang *et al.* 2010](#_ENREF_2_41); [Scheller *et al.* 2012](#_ENREF_2_36); [Post & Pastor 2013](#_ENREF_2_31); [US NERC 2015](#_ENREF_2_39)) and empirical data. For the most abundant species in the New Forest (*Betula pendula*, *Fagus sylvatica*, *Ilex aquifolium*, *Pinus sylvestris*, *Quercus robur*, *Picea abies*, and *Pseudotsuga menziesii*), coarse roots, fine roots, leaf and litter samples were collected and sent to the laboratory (Forest Research, Alice Holt Lodge, Farnham, Surrey) for analysis of their C:N ratios. Sample collection and nutrient analysis was conducted following laboratory protocols (ISO standards 10694:1995 and 13878:1998). Functional group inputs included an additional 17 parameters that were estimated from the literature ([Metherell *et al.* 1993](#_ENREF_2_22); [Scheller *et al.* 2012](#_ENREF_2_36)) and then adjusted during the calibration following the methods outlined in [Scheller *et al.* (2015)](#_ENREF_2_37).

The landscape was divided in 25 ecoregions based on [Newton *et al.* (2013)](#_ENREF_2_26). Ecoregion inputs included nine parameters relating to the initial total C, total N and mineral N, 15 soil properties and the latitude of the study area. For the initial soil conditions (except mineral N) and three of the soil properties (soil depth, percent clay and sand), soil and bulk density samples (25 in the organic layer and 25 in the mineral layer) were collected and sent to the laboratory to be analysed for total C, total N and particle size analysis. Samples collection and soil analysis were conducted following laboratory protocols (ISO standards 10694:1995 and 13878:1998 for C and N, ISO 11272:1998 for bulk density and laser diffraction for particle size analysis). Total C and N were then partitioned into the soil organic matter (SOM) pools (fast, slow, passive) using the equations in [NREL (2000a)](#_ENREF_2_27). Separate soil samples (25 in the organic layer and 25 in the mineral layer) were collected for mineral N (NO3^-^ and NH4^+^) analysis. This included five standard laboratory stages: extraction with 1 Molar potassium chloride (KCl), placing of the vials on a rotary shaker for 30 minutes at 250 rev/min, filtration of the extracts through a Fisher QT 210 filter paper, analysis for NO3^-^ and NH4^+^ by colorimetry using the salicylate-nitroprusside method for NH_4_^+^ and the vanadium method for NO_3_, and conversion of the NO3^-^ and NH4^+^ concentrations to quantities per m^2^ using the bulk density of the soil ([Mulvaney 1996](#_ENREF_2_24); [Miranda *et al.* 2001](#_ENREF_2_23)). Field capacity and wilting point were calculated using the CENTURY soil calculator ([NREL 2000b](#_ENREF_2_28)). Storm flow fraction, base flow fraction, and drain (parameters determining the amount of water runoff and leaching) were extracted from the literature ([Livingston *et al.* 1988](#_ENREF_2_17); [NREL 2000a](#_ENREF_2_27)). The remaining seven soil properties were estimated following the procedure outlined in [Scheller *et al.* (2015)](#_ENREF_2_37) and then adjusted during the calibration.

Baseline climate data required by CENTURY were obtained from monthly means for 1957-2014 measured at Hurn climate station (approx. 10 km from the New Forest boundary) from the UK Met Office ([Met Office 2015](#_ENREF_2_21)).

*Model limitations*

As with any other ecological modelling study, the research presented here is subject to a number of limitations ([Cantarello *et al.* 2011](#_ENREF_2_4)), and these should clearly be borne in mind when interpreting the results. Ideally, model calibration and validation would proceed in a linear fashion whereby a model is fully calibrated and then validated against independent empirical data, an approach that is rarely achieved in practice ([Perera *et al.* 2015](#_ENREF_2_30)). We attempted to address these limitations by identifying those empirical input data for which we had the highest confidence, keeping these constant and adjusting few model parameters to achieve the results that would reflect empirical data. CENTURY calibration followed the procedures outlined in [Scheller *et al.* (2011a)](#_ENREF_2_34) and [Loudermilk *et al.* (2013)](#_ENREF_2_18). We calibrated modelled monthly NEE against measured NEE at Alice Holt flux tower (51º9’13’’ N 0º51’30’’ W) ([Wilkinson *et al.* 2012, updated with the later years](#_ENREF_2_42)) by simulating a single cell having similar species composition, age structure and soil type as present around the flux tower (M. Wilkinson, personal communication). The regression between measured and modelled NEE yielded a significant relationship (*p* < 0.001 both slope and intercept) with R^2^ of 0.77. We also calibrated AGB, so that the mean initial simulated AGB was within 10% of measured data ([Cantarello & Newton 2008](#_ENREF_2_3); [Newton *et al.* 2013](#_ENREF_2_26)) and the accumulation rate was realistic compared to empirical studies ([Keith *et al.* 2009](#_ENREF_2_15); [Evans *et al.* in press](#_ENREF_2_10)). Mean simulated AGB at the end of the simulation and mean measured AGB for the intact forest stands of the dieback gradient in [Evans *et al.* (in press)](#_ENREF_2_10) were 391 and 396 (Mg ha^-1^), respectively. We adjusted the N deposition slope and intercept until the N deposition was within the range observed for the New Forest (~ 17 kg N ha^-1^ yr^-1^; DEFRA, 2011). We also adjusted the decay rates of the SOM pools such that the initial simulated total C and N was within 10% of our empirical data.

Our study might also suffer from the fact that it does not include other important factors that may interact with browsing, wind and diseases, such as drought, insect outbreaks or atmospheric CO_2_, all of which could influence tree mortality. Greater accuracy could be achieved by using the wind, biological disturbance agent and climate extensions of LANDIS-II (www.landis-ii.org), all of which require their own parameterisation and calibration which was beyond the scope of this study. Our aim was not to consider all of the possible disturbances types but instead to provide a novel quantitative assessment of forest resilience, simulating both pulse (sudden disturbance) and press (sustained disturbance) dynamics and focusing on three measurable elements of resilience: resistance, recovery and net change.

References

Blumstein, M. & Thompson, J.R. (2015) Land-use impacts on the quantity and configuration of ecosystem service provisioning in Massachusetts, USA. *Journal of Applied Ecology,* **52,** 1009-1019.

Burns, R.M. & Honkala, B.H. (1990) *Silvics of North America: 1. Conifers; 2. Hardwoods. Agriculture Handbook No. 654*. USDA Forest Service, Washington, D.C.

Cantarello, E. & Newton, A.C. (2008) Identifying cost-effective indicators to assess the conservation status of forested habitats in Natura 2000 sites. *Forest Ecology and Management,* **256,** 815-826.

Cantarello, E., Newton, A.C., Hill, R.A., Tejedor-Garavito, N., Williams-Linera, G., Lopez-Barrera, F., Manson, R.H. & Golicher, D.J. (2011) Simulating the potential for ecological restoration of dryland forests in Mexico under different disturbance regimes. *Ecological Modelling,* **222,** 1112-1128.

DEFRA (2011) UK Deposition Data 2011 [Online]. Department for Environment, Food & Rural Affairs (DEFRA): London, UK. Available: <http://pollutantdeposition.defra.gov.uk/data> [Accessed 19/05/2015].

Di Febbraro, M., Roscioni, F., Frate, L., Carranza, M.L., De Lisio, L., De Rosa, D., Marchetti, M. & Loy, A. (2015) Long-term effects of traditional and conservation-oriented forest management on the distribution of vertebrates in Mediterranean forests: a hierarchical hybrid modelling approach. *Diversity and Distributions,* **21,** 1141-1154.

Duveneck, M.J. & Scheller, R.M. (2015) Climate-suitable planting as a strategy for maintaining forest productivity and functional diversity. *Ecological Applications,* **25,** 1653-1668.

Duveneck, M.J., Scheller, R.M. & White, M.A. (2014) Effects of alternative forest management on biomass and species diversity in the face of climate change in the northern Great Lakes region (USA). *Canadian Journal of Forest Research,* **44,** 700-710.

Escudero, A., Delarco, J.M., Sanz, I.C. & Ayala, J. (1992) Effects of Leaf Longevity and Retranslocation Efficiency on the Retention Time of Nutrients in the Leaf Biomass of Different Woody Species. *Oecologia,* **90,** 80-87.

Evans, P.M., Newton, A.C., Cantarello, E., Martin, P.A., Sanderson, N., Jones, N., Barsoum, N., Cottrell, J.E., A'Hara, S.W. & Fuller, L. (in press) Thresholds of biodiversity and ecosystem function in a forest ecosystem undergoing dieback. *Scientific Reports*.

Gustafson, E.J., De Bruijn, A.M.G., Pangle, R.E., Limousin, J.-M., McDowell, N.G., Pockman, W.T., Sturtevant, B.R., Muss, J.D. & Kubiske, M.E. (2015) Integrating ecophysiology and forest landscape models to improve projections of drought effects under climate change. *Global Change Biology,* **21,** 843-856.

He, H.S. & Mladenoff, D.J. (1999) Spatially explicit and stochastic simulation of forest-landscape fire disturbance and succession. *Ecology,* **80,** 81-99.

Hill, M.O., Preston, C.D. & Roy, D.B. (2004) *PLANTATT - attributes of British and Irish plants: status, size, life history, geography and habitats*. Centre for Ecology and Hydrology, Cambridgeshire.

Ishii, H., Yoshimura, K. & Mori, A. (2009) Convergence of leaf display and photosynthetic characteristics of understory Abies amabilis and Tsuga heterophylla in an old-growth forest in southwestern Washington State, USA. *Tree Physiology,* **29,** 989-998.

Keith, H., Mackey, B.G. & Lindenmayer, D.B. (2009) Re-evaluation of forest biomass carbon stocks and lessons from the world's most carbon-dense forests. *Proceedings of the National Academy of Sciences,* **106,** 11635-11640.

Kretchun, A.M., Scheller, R.M., Lucash, M.S., Clark, K.L., Hom, J. & Van Tuyl, S. (2014) Predicted Effects of Gypsy Moth Defoliation and Climate Change on Forest Carbon Dynamics in the New Jersey Pine Barrens. *Plos One,* **9**.

Livingston, E., McCarron, E., Cox, J., Sanzone, P., Nonpoint Source Management Section & Florida Department of Environmental Regulation (1988) Calculations to estimate runoff. *The Florida Development Manual. A Guide to Sound Land and Water Management*. Florida Department of Environmental Regulation, Tallahassee, Florida.

Loudermilk, E.L., Scheller, R.M., Weisberg, P.J., Yang, J., Dilts, T.E., Karam, S.L. & Skinner, C. (2013) Carbon dynamics in the future forest: the importance of long-term successional legacy and climate–fire interactions. *Global Change Biology,* **19,** 3502-3515.

Lucash, M.S., Scheller, R.M., Kretchun, A.M., Clark, K.L. & Hom, J. (2014) Impacts of fire and climate change on long-term nitrogen availability and forest productivity in the New Jersey Pine Barrens. *Canadian Journal of Forest Research,* **44,** 404-412.

Mediavilla, S. & Escudero, A. (2003) Leaf life span differs from retention time of biomass and nutrients in the crowns of evergreen species. *Functional Ecology,* **17,** 541-548.

Met Office (2015) UK climate - Historic station data [Online]. Exeter, UK. Available: <http://www.metoffice.gov.uk/pub/data/weather/uk/climate/stationdata/hurndata.txt> [Accessed 08/12/2016].

Metherell, A.K., Harding, L.A., Cole, C.V. & Parton, W.J. (1993) CENTURY Soil Organic Matter Model Environment. Technical Documentation Agroecosystem Version 4.0. GPSR Technical Report No. 4. United States Department of Agriculture, Agricultrual Research Service and Great Plains Systems Research Unit, Colorado, USA.

Miranda, K.M., Espey, M.G. & Wink, D.A. (2001) A Rapid, Simple Spectrophotometric Method for Simultaneous Detection of Nitrate and Nitrite. *Nitric Oxide,* **5,** 62-71.

Mulvaney, R.L. (1996) Nitrogen - inorganic forms. *Methods of Soil Analysis. Part 3. Chemical Methods* (ed. D.L. Sparks), pp. 1123-1184. Soil Science Society of America, Madison, WI, USA.

Newton, A. & Cantarello, E. (2015) Restoration of forest resilience: An achievable goal? *New Forests***,** 1-24.

Newton, A.C., Cantarello, E., Tejedor, N. & Myers, G. (2013) Dynamics and Conservation Management of a Wooded Landscape under High Herbivore Pressure. *International Journal of Biodiversity,* **2013,** 15.

NREL (2000a) CENTURY Parameterization Workbook. Natural Resource Ecology Laboratory (NREL), Colorado, USA.

NREL (2000b) Soil calculator [Online]. Natural Resource Ecology Laboratory (NREL): Colorado, USA. Available: https://[www.nrel.colostate.edu/projects/century/](http://www.nrel.colostate.edu/projects/century/) [Accessed 19/05/15].

Pauli, B.P., Zollner, P.A., Haulton, G.S., Shao, G. & Shao, G. (2015) The simulated effects of timber harvest on suitable habitat for Indiana and northern long-eared bats. *Ecosphere,* **6,** 1-24.

Perera, A.H., Sturtevant, B.R. & Buse, L.J. (2015) *Simulation Modeling of Forest Landscape Disturbances*. Springer International Publishing, Switzerland.

Post, W.M. & Pastor, J. (2013) LINKAGES: An Individual-based Forest Ecosystem Biogeochemistry Model. ORNL Distributed Active Archive Center. Available: <http://dx.doi.org/10.3334/ORNLDAAC/1166> [Accessed 25/01/2016].

Pyatt, G., Spencer, J., Hutchby, L., Davani, S., Flethcher, J. & Purdy, K. (2003) *Applying the ecological site classification in the Lowlands. Technical paper 33*. Forestry Commission, Edinburgh.

Reich, P.B., Oleksyn, J., Modrzynski, J. & Tjoelker, M.G. (1996) Evidence that longer needle retention of spruce and pine populations at high elevations and high latitudes is largely a phenotypic response. *Tree Physiology,* **16,** 643-647.

Scheller, R., Van Tuyl, S., Clark, K., Hom, J. & La Puma, I. (2011a) Carbon Sequestration in the New Jersey Pine Barrens Under Different Scenarios of Fire Management. *Ecosystems,* **14,** 987-1004.

Scheller, R.M., Hua, D., Bolstad, P.V., Birdsey, R.A. & Mladenoff, D.J. (2011b) The effects of forest harvest intensity in combination with wind disturbance on carbon dynamics in Lake States Mesic Forests. *Ecological Modelling,* **222,** 144-153.

Scheller, R.M., Kretchun, A.M., Van Tuyl, S., Clark, K.L., Lucash, M.S. & Hom, J. (2012) Divergent carbon dynamics under climate change in forests with diverse soils, tree species, and land use histories. *Ecosphere,* **3,** art110.

Scheller, R.M., Lucash, M.S., Creutzburg, M. & Loudermilk, E.L. (2015) *LANDIS-II Century Succession v4.0 Extension User Guide*. Portland State University, Portland, USA.

Sjöström, E. (1993) *Wood Chemistry: Fundamentals and Applications*. Academic Press, California, USA.

US NERC (2015) Foliar chemistry database. US Forest Service Northeastern Research Station (NERC) and University of New Hampshire Complex Systems Research Cente. Available: <http://www.folchem.sr.unh.edu> [Accessed 13/05/2015].

Wang, F., Mladenoff, D.J., Forrester, J.A., Blanco, J.A., Scheller, R.M., Peckham, S.D., Keough, C. & Lucash, M. (2014) Multi-model simulations of forest harvesting effects on long-term productivity and CN cycling in aspen forests. *Ecological Applications,* **24,** 1374-1389.

Wang, Y., Gril, J., Clair, B., Minato, K. & Sugiyama, J. (2010) Wood properties and chemical composition of the eccentric growth branch of Viburnum odoratissimum var. awabuki. *Trees,* **24,** 541-549.

Wilkinson, M., Eaton, E.L., Broadmeadow, M.S.J. & Morison, J.I.L. (2012) Inter-annual variation of carbon uptake by a plantation oak woodland in south-eastern England. *Biogeosciences,* **9,** 5373-5389.

### **Appendix S3.** Detailed methods for the ecosystem services and biodiversity values obtained by field surveys.

Twelve sites where recent dieback of broadleaved woodlands has been observed were selected for study. Within each site, five 20 x 20 m survey plots were established along a gradient of woodland dieback, using basal area (BA) as a measure of forest structure. Plots were situated to provide values of 100%, 75%, 50%, 25% and 0% BA, with 100% representing an intact forest stand and 0% indicating complete death of all canopy trees (Table S3-1).

**Table S3-1.** Basal area (BA) statistics. Mean, standard deviation (SD) of BA for each of the stages of dieback.

| Stage of dieback | N | Mean BA | SD BA |
| --- | --- | --- | --- |
| 100% - intact forest | 12 | 66.42 | 10.29 |
| 75% - slight dieback | 12 | 49.71 | 1.36 |
| 50% - moderate dieback | 12 | 33.37 | 1.79 |
| 25% - major dieback | 12 | 17.45 | 1.47 |
| 0% - total dieback | 12 | 0 | 0 |

Detailed searches of each plot were undertaken to identify species richness of commercially harvested and ectomycorrhizal fungi, ground flora, and epiphytic lichens. In each plot, net N mineralisation was recorded in-situ following [DeLuca](#_ENREF_3_2) *[et al.](#_ENREF_3_2)* [(2013)](#_ENREF_3_2). A polyester mesh ionic resin capsule (Unibest, Walla Walla, WA, USA) was buried in the centre of each plot, 10 cm deep into the mineral layer and removed from the ground four months later. Soil respiration rate was measured using a portable EGM-4 Environmental Gas Monitor CO_2_ infrared gas analyzer (IRGA) equipped with a closed system soil respiration chamber (PP Systems, Amesbury, MA, USA). All measurements were recorded between 10:00 am and 14:00 pm on sunny days within a month of each other. After automatic flushing and calibration of the chamber, the chamber was inserted 2 cm into the soil after any vegetation had been removed from the surface. The CO_2_ concentration was measured continuously for 2 minutes. Five measurements were taken from each survey plot and then averaged to produce a mean soil respiration rate for the whole plot.

Recreation and aesthetic values were measured by conducting a questionnaire survey of 200 visitors distributed equally across ten car parks of the SSSI New Forest boundary. Visitors were asked to score a series of photo-realistic images for recreation and aesthetic value separately, using a Likert-type scale (1-5) ([Gosal 2016](#_ENREF_3_3)). The photo-realistic images represented 100%, 75%, 50%, 25% and 0% BA (Figure S3-1), and were manipulated from a base image to ensure that visitors were assessing woodland cover, rather than any other landscape feature, following [Ode *et al.* (2008)](#_ENREF_3_4) and [Cordingley (2012)](#_ENREF_3_1). Specifically, the base image was derived from a range of photos taken in Ridley Wood (New Forest) at varying distances from the beech woodland. The different BA percentages were created by clipping sections of tree stands from the base image. To maintain realism, the edges of the trees were ‘feathered’ and the gaps between trees filled with a standardised background. The outline of the tree stands in each image was manipulated to look uneven and not repetitive.

| (a)  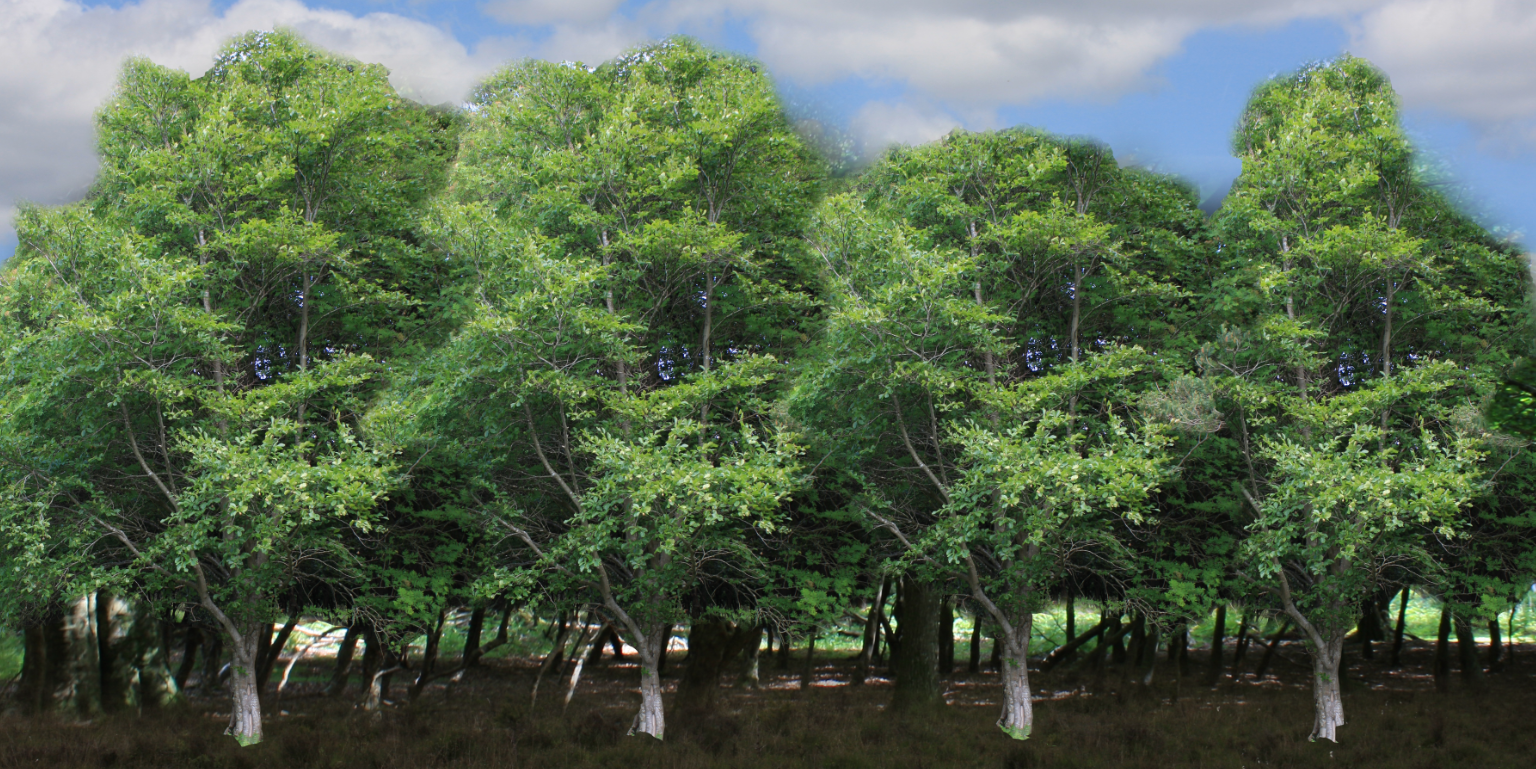 | (b)  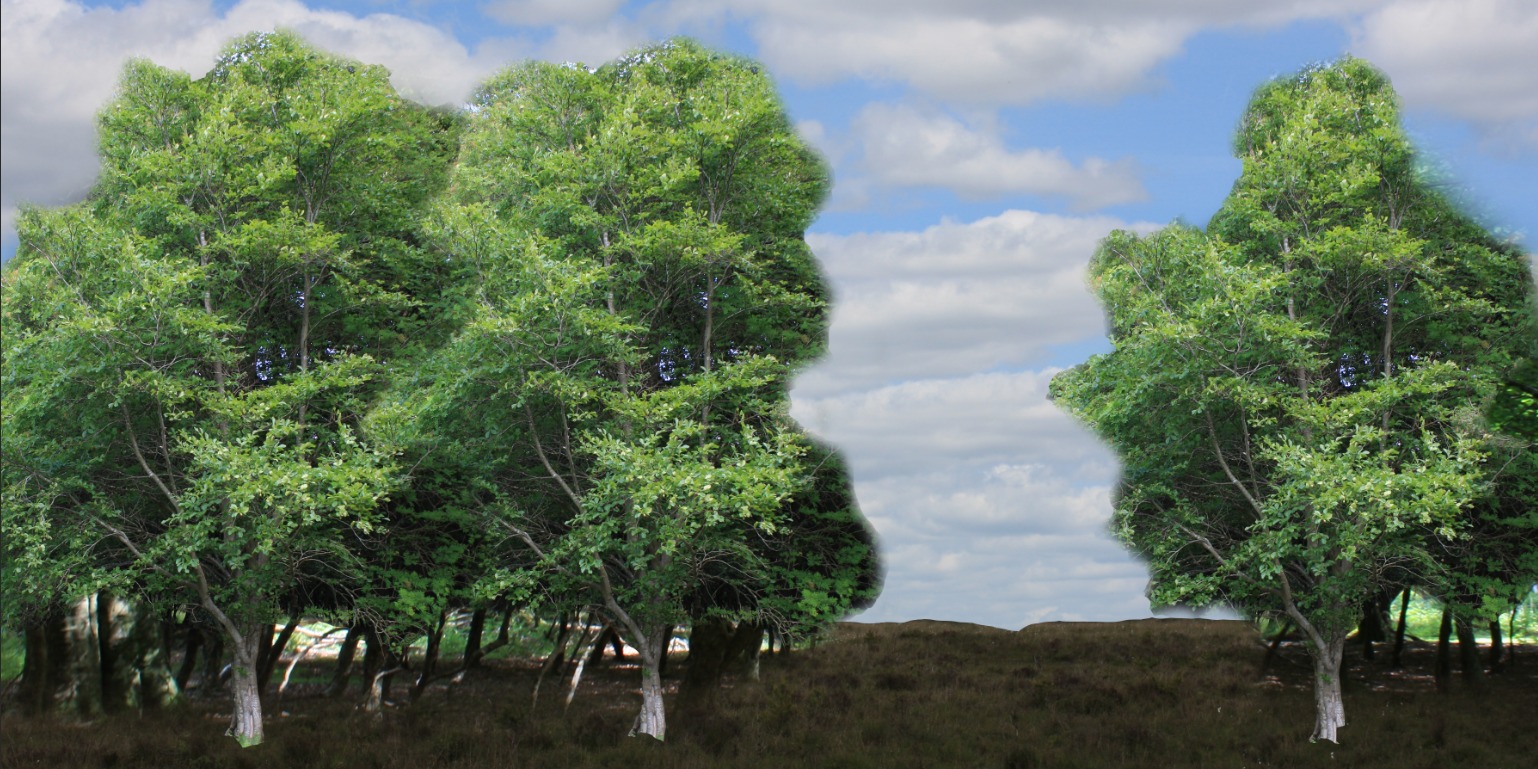 |
| --- | --- |
| (c)  **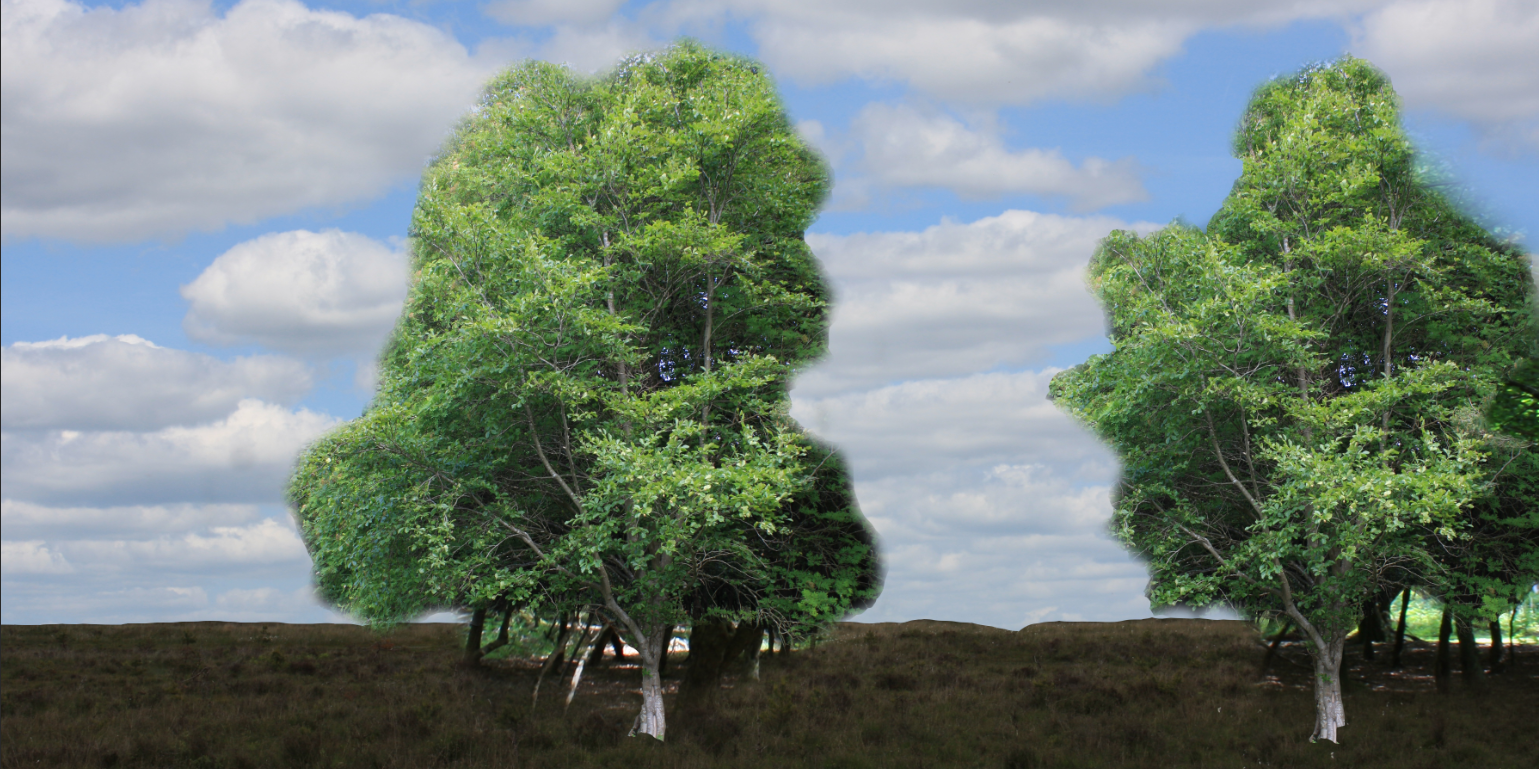** | (d)  **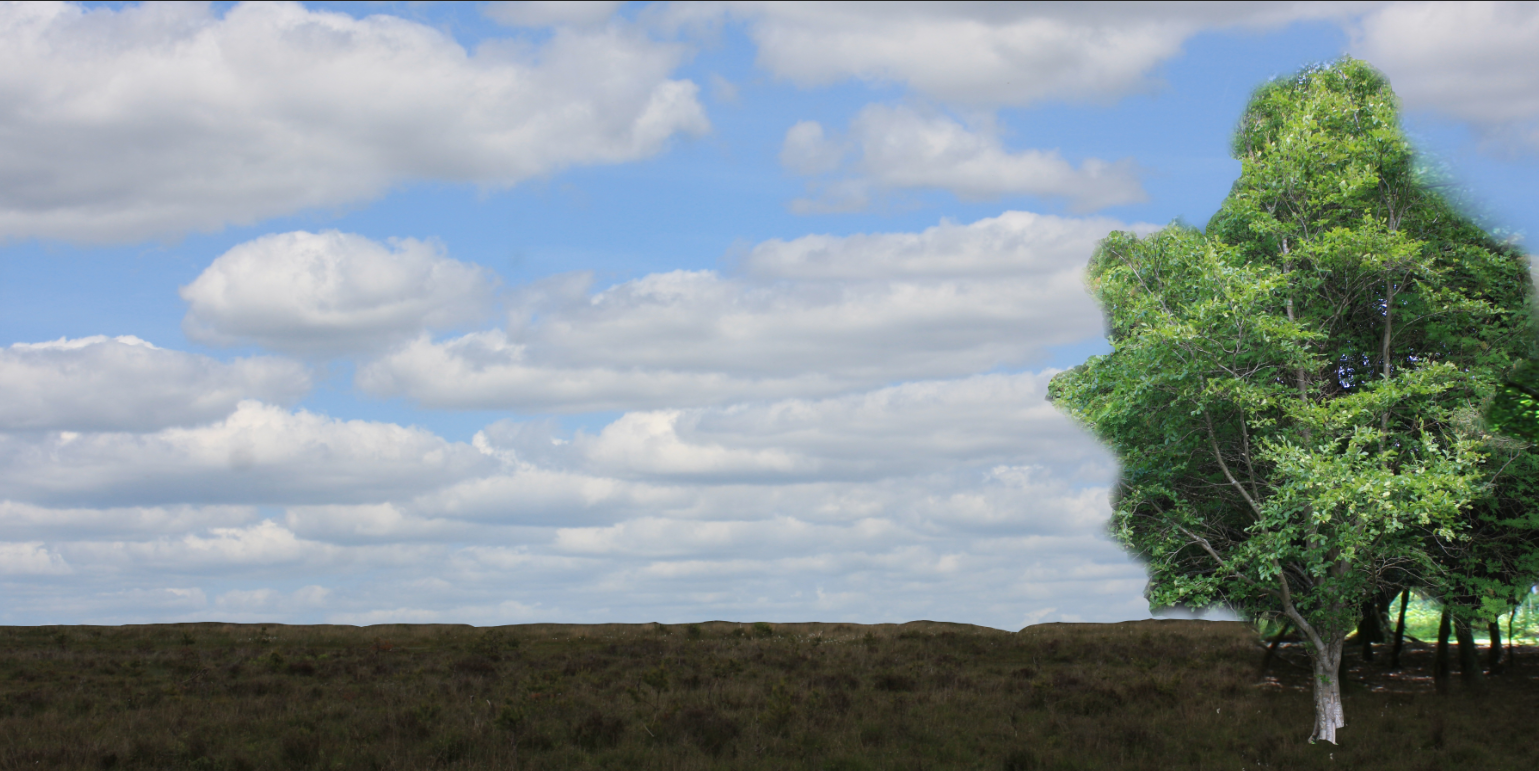** |
| (e) **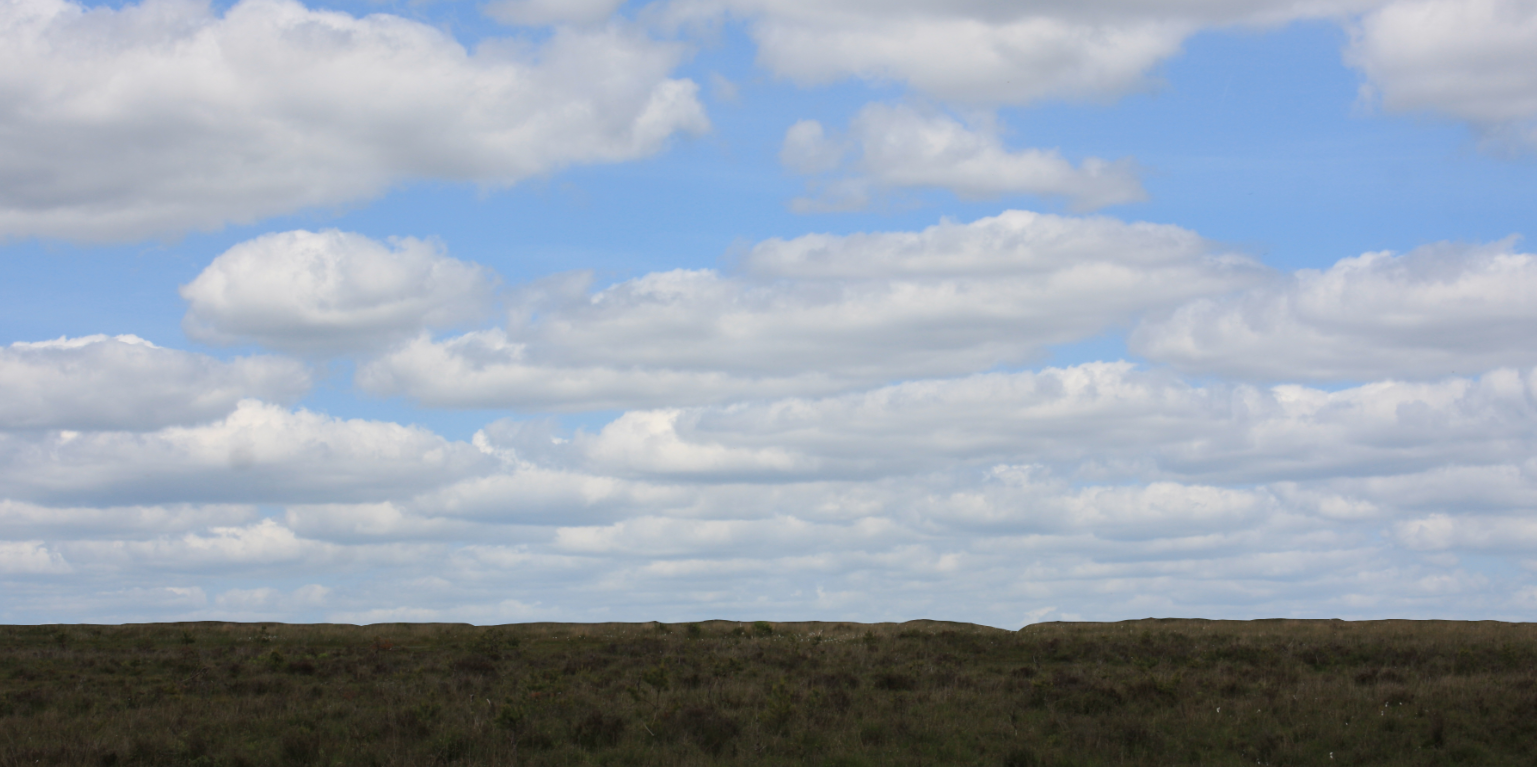** |  |

**Figure S3-1.** Photorealistic images used to elicit recreation and aesthetic values: (a) 100% BA, (b) 75% BA, (c) 50% BA, (d) 25% BA (e) 0% BA.

References

Cordingley, J.E. (2012) Ecosystem Service Provision in Dynamic Heath Landscapes. Doctor of Philosophy, Bournemouth University.

DeLuca, T.H., Zewdie, S.A., Zackrisson, O., Healey, J.R. & Jones, D.L. (2013) Bracken fern (Pteridium aquilinum L. kuhn) promotes an open nitrogen cycle in heathland soils. *Plant and Soil,* **367,** 521-534.

Gosal, A. (2016) A multifaceted approach to spatial analysis of ecosystem services: a case study in the New Forest National Park. Doctor of Philosophy, Bournemouth University.

Ode, A., Tveit, M.S. & Fry, G. (2008) Capturing landscape visual character using indicators: Touching base with landscape aesthetic theory. *Landscape Research,* **33,** 89-117.

### **Appendix S4.** Details for the linear mixed models (LMMs) fitted to estimate the relationships between aboveground biomass (AGB), and the ecosystem services and biodiversity values (Y variables).

**Table S4-1.** LMMs fitted and associated measures of parsimony (AICc), support (ΔAICc, AICc weight) and goodness of fit (Marginal R^2^). Site was used as random effect.

| Y Variable | Model structure | Log likelihood | AICc | ΔAICc | AICc weight | Marginal R^2^ |
| --- | --- | --- | --- | --- | --- | --- |
| Aesthetic value | Linear | -2124.17 | 4256.38 | 0 | 1 | 0.14 |
|  | Quadratic | -2135.89 | 4281.84 | 25.46 | 0 | 0.14 |
|  | Null | -2249.16 | 4504.35 | 247.97 | 0 | 0 |
| Commercially harvested fungi richness | Linear | -58.42 | 123.28 | 0 | 0.71 | 0.19 |
|  | Quadratic | -58.21 | 125.15 | 1.87 | 0.28 | 0.23 |
|  | Null | -64.03 | 132.28 | 9 | 0.01 | 0 |
| Ectomycorrhizal fungi richness | Quadratic | -151 | 310.75 | 0 | 0.85 | 0.5 |
|  | Linear | -153.91 | 314.26 | 3.51 | 0.15 | 0.42 |
|  | Null | -183.05 | 370.32 | 59.57 | 0 | 0 |
| Epiphytic lichens richness | Quadratic | -241.98 | 492.69 | 0 | 0.9 | 0.21 |
|  | Linear | -245.32 | 497.08 | 4.39 | 0.1 | 0.15 |
|  | Null | -261.67 | 527.55 | 34.86 | 0 | 0 |
| Ground flora richness | Linear | -194.62 | 395.67 | 0 | 0.74 | 0.47 |
|  | Quadratic | -194.53 | 397.79 | 2.12 | 0.26 | 0.46 |
|  | Null | -253.34 | 510.89 | 115.22 | 0 | 0 |
| Net N mineralisation | Quadratic | -239.73 | 490.72 | 0 | 0.63 | 0.05 |
|  | Linear | -241.57 | 491.96 | 1.24 | 0.34 | 0.05 |
|  | Null | -245.1 | 496.69 | 5.97 | 0.03 | 0 |
| Recreation value | Linear | -2152.12 | 4312.28 | 0 | 1 | 0.03 |
|  | Quadratic | -2162.23 | 4334.53 | 22.25 | 0 | 0.03 |
|  | Null | -2170.47 | 4346.97 | 34.69 | 0 | 0 |
| Soil respiration rate | Linear | -68.52 | 145.77 | 0 | 0.61 | 0.1 |
|  | Null | -70.24 | 146.91 | 1.14 | 0.34 | 0 |
|  | Quadratic | -69.89 | 150.9 | 5.13 | 0.05 | 0.11 |

**Table S4-2.** Model-averaged coefficients of the LMMs fitted. Model averaging was undertaken with AICc weights used to calculate coefficients and associated standard errors (SE) and *P* values using the MuMIn package ([Barton 2015](#_ENREF_4_1)).

| Y Variable | Model parameter | Estimate | SE | *P* value |
| --- | --- | --- | --- | --- |
| Aesthetic values | Intercept | -0.263 | 0.147 | 0.074 |
|  | AGB -linear | 0.007 | 0.01 | <0.001 |
|  | AGB - quadratic | <0.001 | <0.001 | 1 |
| Commercially harvested fungi richness | Intercept | -0.52 | 0.22 | 0.019 |
|  | AGB -linear | 0.53 | 0.18 | 0.004 |
|  | AGB - quadratic | -0.02 | 0.07 | 0.771 |
| Ectomycorrhizal fungi richness | Intercept | 2.15 | 0.08 | <0.001 |
|  | AGB -linear | 0.39 | 0.06 | <0.001 |
|  | AGB - quadratic | -0.07 | 0.05 | 0.112 |
| Epiphytic lichens richness | Intercept | 3.47 | 0.05 | <0.001 |
|  | AGB -linear | 0.16 | 0.03 | <0.001 |
|  | AGB - quadratic | -0.05 | 0.03 | 0.064 |
| Ground flora richness | Intercept | 2.63 | 0.08 | <0.001 |
|  | AGB -linear | -0.39 | 0.04 | <0.001 |
|  | AGB - quadratic | <0.001 | 0.02 | 0.843 |
| Net N mineralisation | Intercept | 20.79 | 6.15 | 0.001 |
|  | AGB -linear | -4.97 | 3.15 | 0.123 |
|  | AGB - quadratic | -0.4 | 1.96 | 0.843 |
| Recreation value | Intercept | 0.558 | 0.141 | <0.001 |
|  | AGB -linear | 0.003 | <0.001 | <0.001 |
|  | AGB - quadratic | <0.001 | <0.001 | 0.997 |
| Soil respiration rate | Intercept | 2.3 | 1.67 | 0.168 |
|  | AGB -linear | 0.16 | 0.14 | 0.256 |
|  | AGB - quadratic | 0 | 0.02 | 0.901 |

References

Barton, K. (2015) *MuMIn: Multi-model Inference*.

### **Appendix S5.** Details for the linear mixed models (LMMs) fitted to estimate the relationships between resistance, recovery time, net change, and the degree of disturbance for each of the ecosystem services and biodiversity values.

**Table S5-1.** Details for the linear mixed models (LMMs) fitted to estimate the relationships between resistance and the degree of disturbance for each of the ecosystem services and biodiversity values. Difference between pulse and pulse+press values of resistance were tested using the Wilcoxon signed rank test.

|  | Pulse set (P) | | | | | | Pulse + press set (PP) | | | | | | Threshold? | | Difference between pulse and pulse+press? | |
| --- | --- | --- | --- | --- | --- | --- | --- | --- | --- | --- | --- | --- | --- | --- | --- | --- |
| Resistance for | Best model | df | AICc | Intercept | Slope | R^2^ | Best model | df | AICc | Intercept | Slope | R^2^ | P | PP | *P* (est.) |  |
| Aboveground biomass | Linear | 4 | -35.07 | 0.521 | -0.297 | 0.98 | Linear | 4 | -31.21 | 0.529 | -0.304 | 0.97 | No | No | 0.410 | No |
| Aesthetic values | Linear | 4 | -96.93 | 0.912 | -0.064 | 0.99 | Linear | 4 | -94.32 | 0.914 | -0.065 | 0.99 | No | No | 0.349 | No |
| Commercially harvested fungi richness | Linear | 4 | -33.16 | 0.598 | -0.236 | 0.96 | Linear | 4 | -29.8 | 0.604 | -0.242 | 0.95 | No | No | 0.514 | No |
| Ectomycorrhizal fungi richness | Linear | 4 | -55.71 | 0.681 | -0.21 | 0.99 | Linear | 4 | -49.84 | 0.686 | -0.214 | 0.98 | No | No | 0.410 | No |
| Epiphytic lichens richness | Linear | 4 | -71.29 | 0.844 | -0.118 | 0.98 | Linear | 4 | -69.79 | 0.847 | -0.119 | 0.98 | No | No | 0.268 | No |
| Ground flora richness | Null | 3 | -1206.55 | 1 | NA | 0 | Null | 3 | -71.6 | 0.991 | NA | 0 | No | No | 0.181 | No |
| Net N mineralisation | Null | 3 | -1206.55 | 1 | NA | 0 | Null | 3 | -83.27 | 0.993 | NA | 0 | No | No | 0.181 | No |
| Recreation value | Linear | 4 | -71.39 | 0.812 | -0.138 | 0.99 | Linear | 4 | -68.62 | 0.816 | -0.139 | 0.99 | No | No | 0.293 | No |
| Soil nitrogen stock | Linear | 4 | -102.06 | 0.988 | -0.013 | 0.83 | Linear | 4 | -103.15 | 0.988 | -0.013 | 0.84 | No | No | 0.286 | No |
| Soil respiration rate | Linear | 4 | -116.6 | 0.943 | -0.04 | 0.99 | Linear | 4 | -111.37 | 0.944 | -0.04 | 0.99 | No | No | 0.379 | No |
| Timber volume | Linear | 4 | -23.51 | 0.428 | -0.335 | 0.96 | Linear | 4 | -21.85 | 0.438 | -0.343 | 0.96 | No | No | 0.514 | No |
| Total carbon stock | Linear | 4 | -71.09 | 0.775 | -0.154 | 0.99 | Linear | 4 | -63.18 | 0.778 | -0.157 | 0.99 | No | No | 0.410 | No |
| Tree species richness | Null | 3 | -1206.55 | 1 | NA | 0 | Null | 3 | -1206.55 | 1 | NA | 0 | No | No | 1.000 | No |

**Table S5-2.** Details for the linear mixed models (LMMs) fitted to estimate the relationships between recovery time and the degree of disturbance for each of the ecosystem services and biodiversity values. Difference between pulse and pulse+press values of resistance were tested using the Wilcoxon signed rank test. For this analysis only recovery times ≤ 100 years were considered, corresponding to a degree of disturbance ≤ 60% (see Figure 5).

|  | Pulse set (P) | | | | | | Pulse + press set (PP) | | | | | | Threshold? | | Difference between pulse and pulse+press? | |
| --- | --- | --- | --- | --- | --- | --- | --- | --- | --- | --- | --- | --- | --- | --- | --- | --- |
| Recovery time for | Best model | df | AICc | Intercept | Slope | R^2^ | Best model | df | AICc | Intercept | Slope | R^2^ | P | PP | *P* (est.) |  |
| Aboveground biomass | Linear | 4 | 83.09 | 3.567 | 0.745 | 0.93 | Linear | 4 | 103.7 | -4.8 | 1.577 | 0.9 | No | No | 0.001 | Yes |
| Aesthetic values | Linear | 4 | 82.33 | 3.7 | 0.727 | 0.94 | Linear | 4 | 104.69 | -5.2 | 1.565 | 0.89 | No | No | 0.002 | Yes |
| Commercially harvested fungi richness | Linear | 4 | 83.34 | 3.7 | 0.777 | 0.94 | Linear | 4 | 102.19 | -3.967 | 1.593 | 0.91 | No | No | 0.001 | Yes |
| Ectomycorrhizal fungi richness | Linear | 4 | 82.51 | 3.567 | 0.728 | 0.94 | Linear | 4 | 104.47 | -5.1 | 1.57 | 0.89 | No | No | 0.001 | Yes |
| Epiphytic lichens richness | Linear | 4 | 83.9 | 3.3 | 0.69 | 0.92 | Linear | 4 | 107.14 | -6.133 | 1.543 | 0.86 | No | No | 0.002 | Yes |
| Recreation value | Linear | 4 | 83.75 | 3.433 | 0.705 | 0.92 | Linear | 4 | 106.15 | -5.767 | 1.553 | 0.87 | No | No | 0.002 | Yes |
| Soil nitrogen stock | Null | 3 | 33.27 | 6.333 | NA | 0 | Null | 3 | 33.27 | 6.333 | NA | 0 | No | No | 0.233 | No |
| Soil respiration rate | Linear | 4 | 83.09 | 3.567 | 0.745 | 0.93 | Linear | 4 | 103.7 | -4.8 | 1.577 | 0.9 | No | No | 0.001 | Yes |
| Timber volume | Linear | 4 | 97.58 | 0.6 | 1.547 | 0.94 | Linear | 4 | 104.24 | 0.1 | 1.688 | 0.9 | No | No | 0.236 | No |
| Total carbon stock | Linear | 4 | 82.13 | 3.767 | 0.73 | 0.94 | Linear | 4 | 104.85 | -5.4 | 1.563 | 0.88 | No | No | 0.002 | Yes |

**Table S5-3.** Details for the linear mixed models (LMMs) fitted to estimate the relationships between net change and the degree of disturbance for each of the ecosystem services and biodiversity values. Difference between pulse and pulse+press values of resistance were tested using the Wilcoxon signed rank test. Int, intercept; QC, quadratic coefficient; N, null; L, linear; Q, quadratic.

|  | Pulse set (P) | | | | | |  | Pulse + press set (PP) | | | | | |  | Threshold? | | Difference between pulse and pulse+press? | |
| --- | --- | --- | --- | --- | --- | --- | --- | --- | --- | --- | --- | --- | --- | --- | --- | --- | --- | --- |
| Net change for | Best model | df | AICc | Int | Slope | QC | R^2^ | Best model | df | AICc | Int | Slope | R^2^ | QC | P | PP | *P* (est.) |  |
| Aboveground biomass | N | 3 | -1206.55 | 1 | NA | NA | 0 | Q | 5 | -31.94 | 0.906 | -0.221 | -0.107 | 0.98 | No | Yes | 0.009 | Yes |
| Aesthetic values | N | 3 | -1206.55 | 1 | NA | NA | 0 | Q | 5 | -92.02 | 0.989 | -0.037 | -0.021 | 0.99 | No | Yes | 0.009 | Yes |
| Commercially harvested fungi richness | N | 3 | -1206.55 | 1 | NA | NA | 0 | Q | 5 | -37.18 | 0.912 | -0.187 | -0.084 | 0.98 | No | Yes | 0.009 | Yes |
| Ectomycorrhizal fungi richness | N | 3 | -1206.55 | 1 | NA | NA | 0 | Q | 5 | -48.45 | 0.948 | -0.142 | -0.075 | 0.98 | No | Yes | 0.009 | Yes |
| Epiphytic lichens richness | N | 3 | -1206.55 | 1 | NA | NA | 0 | Q | 5 | -70.16 | 0.983 | -0.065 | -0.039 | 0.98 | No | Yes | 0.009 | Yes |
| Ground flora richness | Q | 5 | -58.76 | 0.591 | 0.121 | 0.023 | 0.98 | Q | 5 | -28.87 | 0.902 | 0.171 | -0.064 | 0.95 | Yes | Yes | <0.001 | Yes |
| Net mineralisation | L | 4 | -62.27 | 0.673 | 0.126 | NA | 0.98 | Q | 5 | -37.4 | 0.929 | 0.147 | -0.065 | 0.96 | No | Yes | <0.001 | Yes |
| Recreation value | N | 3 | -1206.55 | 1 | NA | NA | 0 | Q | 5 | -65.59 | 0.977 | -0.08 | -0.046 | 0.98 | No | Yes | 0.009 | Yes |
| Soil nitrogen stock | N | 3 | -1206.55 | 1 | NA | NA | 0 | N | 3 | -1206.55 | 1 | NA | NA | 0 | No | No | 1.000 | No |
| Soil respiration rate | N | 3 | -1206.55 | 1 | NA | NA | 0 | Q | 5 | -106.79 | 0.992 | -0.024 | -0.013 | 0.99 | No | Yes | 0.009 | Yes |
| Timber volume | Q | 5 | -26.31 | 0.936 | -0.249 | -0.149 | 0.97 | Q | 5 | -8.01 | 0.784 | -0.343 | -0.118 | 0.95 | Yes | Yes | 0.009 | Yes |
| Total carbon stock | N | 3 | -1206.55 | 1 | NA | NA | 0 | Q | 5 | -36.4 | 0.949 | -0.17 | -0.097 | 0.97 | No | Yes | 0.009 | Yes |
| Tree species richness | N | 3 | -110.99 | 0.929 | NA | NA | 0 | N | 3 | -73.4 | 0.852 | NA | NA | 0 | No | No | <0.001 | Yes |

### **Appendix S6.** Spearman’s correlation coefficients and *P* values between resistance, recovery time and net change.

Note – uploaded as excel file
